# Ferroptosis as a Translational Axis in Small Cell Lung Cancer: A Systematic Review of Redox Pathways and Precision Oncology Prospects

**DOI:** 10.32604/or.2025.073045

**Published:** 2026-03-23

**Authors:** Donatella Coradduzza, Anna La Salvia, Giuseppe Fanciulli, Maria Rosaria De Miglio

**Affiliations:** 1 Department of Biomedical Sciences, University of Sassari, Sassari, 07100, Italy; 2 National Center for Drug Research and Evaluation, National Institute of Health (ISS), Rome, 00161, Italy; 3 Department of Medicine, Surgery and Pharmacy, University of Sassari, Sassari, 07100, Italy

**Keywords:** Small cell lung cancer, ferroptosis, lipid peroxidation, glutathione peroxidase 4, solute carrier family 7 member 11, molecular subtypes, immunotherapy, oxidative stress, regulated cell death, translational oncology, systematic review

## Abstract

**Background:**

An increasing number of studies have shown that ferroptosis is related to the initiation and development of small cell lung cancer (SCLC). The systematic review aimed to summarize the characteristics of ferroptosis from its pathogenetic role to translational therapeutic implications in SCLC.

**Methods:**

This systematic review, registered in PROSPERO (CRD420251090058), followed PRISMA 2020 guidelines. Comprehensive research of PubMed, Scopus, and Web of Science was performed for studies published between January 2010 and July 2025 investigating ferroptosis mechanisms, genetic or pharmacological modulation, or molecular profiling in SCLC. Two reviewers independently performed data extraction and quality assessment.

**Results:**

Nineteen preclinical studies met the inclusion criteria. Key regulators included solute carrier family 7 member 11 (SLC7A11), glutathione peroxidase 4 (GPX4), ferroptosis suppressor protein 1 (FSP1), and acyl-CoA synthetase long chain family member 4 (ACSL4). The molecular subtypes of SCLC, achaete-scute homolog 1 (ASCL1), neuronal differentiation 1 (NEUROD1), POU class 2 homeobox 3 (POU2F3), and Yes1 associated transcriptional regulator (YAP1) exhibit differential ferroptosis gene expressions, influencing therapeutic responsiveness. Non-neuroendocrine subtypes are more ferroptosis-prone, whereas neuroendocrine variants display enhanced antioxidant defenses. Ferroptosis induction also promotes immune activation through stimulator of interferon genes (STING)-mediated CD8^+^ T-cell recruitment.

**Conclusions:**

Ferroptosis constitutes a promising therapeutic axis in SCLC. Integrating ferroptosis biomarkers into molecular stratification frameworks could refine patient selection and support precision oncology strategies, warranting further translational and clinical validation.

## 1 Introduction

### 1.1 Clinical and Molecular Landscape of SCLC

Small cell lung cancer (SCLC) is a highly aggressive neuroendocrine malignancy, accounting for approximately 13%–15% of all lung cancer cases with an estimated global incidence of 250,000 new cases each year and at least 200,000 deaths annually [1]. Despite therapeutic advances in recent decades, the prognosis for SCLC patients remains dismal, with a 5-year survival rate of less than 7% due to rapid metastasis and the development of resistance to chemo-immunotherapy [2]. Traditionally, the etoposide-platinum (EP) doublet regimen has been the standard of care in first-line setting treatment for patients with extensive stage (ES) SCLC [2–4]. For patients with limited disease (LD), the combination of concurrent chemotherapy and thoracic radiotherapy has emerged as an effective therapeutic strategy [5]. However, although the initial response rates of up to 80% are achieved with this regimen, the recurrence occurs in the large majority of patients with both LD and ES-SCLC [6,7]. In light of the limited durable responses to platinum-based regimens, the SCLC treatment paradigm has recently shifted significantly with the integration of immune checkpoint inhibitors into first-line platinum-doublet chemotherapy, as demonstrated by the IMpower133, CASPIAN, and CAPSTONE-1 clinical trials [8–10]. Unfortunately, despite demonstrated survival benefits and a well-tolerated safety profile for chemo-immunotherapy compared to chemotherapy alone, only a small percentage of patients with SCLC experience long-lasting benefits, and the magnitude of benefit from immunotherapy in SCLC is considerably more modest than that observed in other malignancies. Consequently, a deeper understanding of molecular profiles and the identification of predictive biomarkers to optimize clinical decision-making have emerged as an unmet clinical need. To date, neither PD-L1 expression nor tumor mutational burden has been established as a reliable predictive factor for response to immune checkpoint inhibitors in ES-SCLC [11]. At the molecular level, it is established that the dual inactivation of the tumor suppressors TP53 and RB1 has a key role in SCLC tumorigenesis, usually targeting achaete-scute homolog 1 (ASCL1)-expressing pulmonary neuroendocrine cells, initiating the cascade necessary for tumor development [12,13]. In contrast, approximately 5% of SCLCs harbour wild-type RB1 and/or TP53 [14]. Significant advances in the understanding of SCLC biology, achieved through the application of multi-omics analyses, have led to a new molecular classification of this cancer [15,16]. This classification identifies four distinct molecular subtypes, based on the expression levels of four transcription factors (TFs): ASCL1 (SCLC-A), Neuronal Differentiation 1 (NEUROD1; SCLC-N), POU Class 2 Homeobox 3 (POU2F3; SCLC-P), and Yes1 Associated Transcriptional Regulator (YAP1; SCLC-Y). It is also important to note that recent terminology includes an ‘inflamed’ SCLC (SCLC-I). The expression of these subtype-specific TFs entails varying levels of neuroendocrine (NE) differentiation, with potential implications for clinical stratification and management. Overall, despite these efforts and the improvements in response rates and survival outcomes, the magnitude of benefit from available treatments for SCLC remains disappointingly low, and a personalized, curative approach for these patients is still lacking.

### 1.2 Ferroptosis: Rationale for Exploration in SCLC

These advances in molecular classification highlight the biological complexity of SCLC. However, they have not yet translated into durable clinical outcomes. This gap underscores the urgent need for novel therapeutic strategies, including those targeting regulated cell death mechanisms such as ferroptosis. Among the emerging regulated cell death pathways, ferroptosis has garnered significant attention due to its potential to overcome chemoresistance in cancers such as SCLC. A substantial body of evidence has demonstrated that tumor growth can be suppressed by inducing ferroptosis. Ferroptosis is an iron-dependent, non-apoptotic form of cell death driven by uncontrolled lipid peroxidation, leading to membrane damage [17]. Due to its distinct biological processes and pathophysiological features, ferroptosis is considered morphologically and biochemically different from apoptosis and necroptosis. Indeed, ferroptotic cells display shrunken mitochondria with reduced cristae and outer mitochondrial membrane rupture, while maintaining an intact plasma membrane, features not observed in apoptosis or necroptosis [17,18]. Apoptosis, in contrast, is characterized by membrane blebbing, nuclear fragmentation, apoptotic body formation, and caspase activation [19]. Necroptosis typically involves cell swelling, early plasma membrane rupture, and activation of the RIPK1–RIPK3–MLKL axis, distinguishing it from ferroptosis. Ferroptosis also differs substantially from pyroptosis, which is defined by inflammasome activation, gasdermin-mediated pore formation, and the release of pro-inflammatory cytokines [20,21]. Morphologically, ferroptosis is characterized by severe mitochondrial shrinkage, increased membrane density, chromatin condensation, and decreased NADH levels. In contrast to other cell death forms, it lacks cell membrane rupture, nuclear atrophy, and swelling of the cytoplasm and organelles [22–24]. Biochemically, ferroptosis involves the depletion of intracellular glutathione (GSH) and reduced activity of GPX4. The consequent failure of GPX4 to reduce lipid peroxides allows Fe^2+^ to oxidize lipids in a Fenton-like reaction, resulting in the massive accumulation of reactive oxygen species (ROS), and ultimately leading to ferroptosis [25–27]. The process of ferroptosis is genetically regulated by a multitude of genes. Ferroptosis has emerged as a promising avenue for cancer therapy. Unlike apoptosis or necrosis, ferroptosis is characterized by the accumulation of reactive ROS and lipid peroxides, leading to irreversible membrane damage [28]. This unique mechanism is particularly relevant for cancers characterized by high oxidative stress and dysregulated iron metabolism, such as SCLC [29]. For these reasons, SCLC, known for its poor prognosis and therapy resistance, may demonstrate heightened susceptibility to ferroptosis-inducing therapies. Furthermore, the induction of ferroptosis can enhance the efficacy of immunotherapy [30], suggesting a complex interplay between the tumor immune microenvironment (TIME) and ferroptotic cell death. This distinct mechanism represents a promising opportunity for developing novel cancer therapies [31]. The therapeutic potential of ferroptosis is being actively explored across a spectrum of malignancies, including non-small cell lung cancer (NSCLC), breast cancer and glioblastoma. Recent evidence further underscores its broad relevance, demonstrating its potent anti-tumor effects in other aggressive carcinomas such as oral cancer [32]. Despite this growing body of evidence in other solid tumors, the role and therapeutic targeting of ferroptosis in SCLC remain systematically unreviewed. This review systematically explores the molecular underpinnings of ferroptosis in SCLC, providing a comprehensive synthesis of preclinical findings, subtype-specific susceptibilities, prognostic gene signatures, and therapeutic implications and associated clinical challenges. This systematic review aims to synthesize the current preclinical evidence on ferroptosis in SCLC, with a focus on molecular regulators, subtype-specific vulnerabilities, prognostic markers, and translational therapeutic implications.

## 2 Materials and Methods

This systematic review was conducted in accordance with the Preferred Reporting Items for Systematic Reviews and Meta-Analyses (PRISMA 2020) guidelines [33]. The PRISMA checklists can be found in the supplementary files-PRISMA checklist and PRISMA abstract checklist. The protocol was prospectively registered in PROSPERO [34] (Registration ID: [CRD420251090058]).

### 2.1 Search Strategy

A systematic literature search was conducted primarily using the following databases, as identified from the included studies: PubMed, Web of Science (WOS), and Scopus. The search covered articles published between January 2010 and July 2025. Keywords and MeSH terms applied included: “ferroptosis”, “small cell lung cancer”, “SCLC”, “GPX4”, “SLC7A11”, “ACSL4”, “biomarkers”, “KEAP1”, “NRF2”, “FSP1”, “lipid peroxidation”, and “iron metabolism”. Additional sources included citation tracking and screening of reference lists from relevant articles to ensure comprehensive inclusion.

### 2.2 Eligibility Criteria (PICO Framework: Population, Intervention, Comparison, Outcome)

Population (P): Human or animal studies investigating SCLC or solid tumors in relation to ferroptosis. Intervention (I): Evaluation of ferroptosis-related biomarkers (e.g., GPX4, SLC7A11, ACSL4, TFRC, FTH1, and NCOA4), pharmacological inducers or inhibitors of ferroptosis. Comparison (C): Standard therapies, placebo, untreated controls, or absence of biomarker expression. Outcomes (O): Primary outcomes included biomarker expression levels, ferroptosis sensitivity, therapeutic response, prognostic value, and translational applicability. Only peer-reviewed original articles were included. Reviews, commentaries, editorials, conference abstracts, and non-English language publications were excluded [35].

### 2.3 Study Selection Process

**Figure 1:**
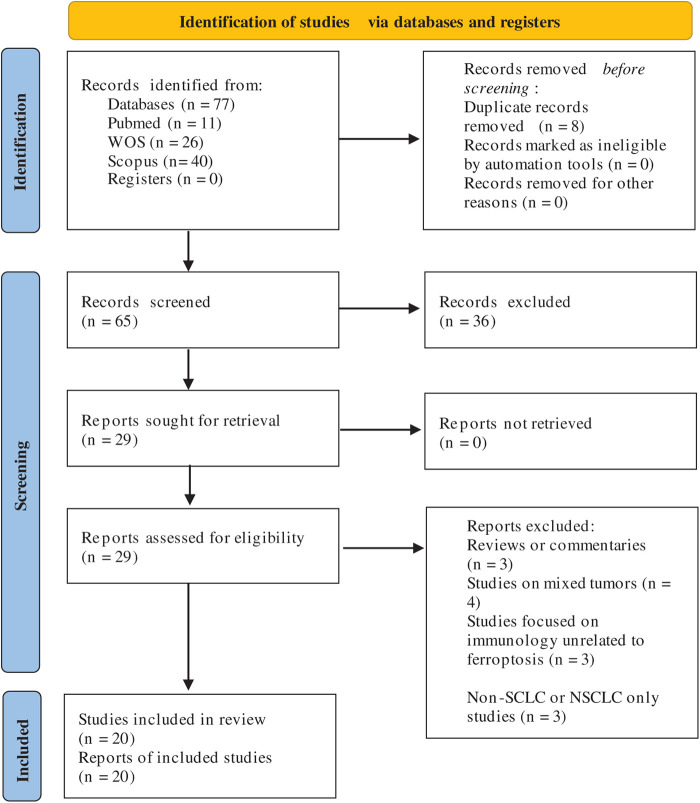
PRISMA flow diagram showing study selection for ferroptosis biomarkers in SCLC [38]

Two independent reviewers screened titles and abstracts for relevance. Full-text articles were then retrieved and assessed against predefined eligibility criteria. Any disagreements between reviewers were resolved through discussion and consensus. A third reviewer adjudicated unresolved disagreements. The detailed study selection process is illustrated in the PRISMA flow diagram (Fig. 1).

### 2.4 Data Extraction and Synthesis

Data were independently extracted by two reviewers using a standardized form, collecting information on study design, sample size, model (human, *in vitro*, *in vivo*), biomarkers analysed, methods of detection, outcomes, and main findings. Extracted data were tabulated and analysed descriptively. For studies with overlapping data, the most comprehensive or recent dataset was retained.

### 2.5 Risk of Bias Assessment

The quality of included studies was assessed using appropriate tools: ROBINS-I for non-randomized studies and RoB-2 for randomized controlled trials [36]. The risk of bias assessment was independently performed by two reviewers.

### 2.6 Certainty of Evidence (Grading of Recommendations, Assessment, Development and Evaluation [GRADE])

The overall certainty of evidence for each outcome was evaluated using the GRADE framework, taking into account risk of bias, inconsistency, indirectness, imprecision, and publication bias [37]. The summary of the GRADE assessment for each included study is presented in Table 1.

**Table 1:** Summary of GRADE assessment for included studies on ferroptosis in small-cell lung cancer (SCLC), detailing risk of bias, precision, indirectness, and overall quality of evidence

Study	Study design	GRADE starting point	Risk of bias	Imprecision	Inconsistency	Indirectness	Publication bias	Other considerations	Final GRADE
Wang et al. [52]	Transcriptomic analysis, clustering	Low (observational)	Moderate: retrospective cohort	Moderate: moderate sample size	Not assessed	Moderate: subtype classification only	Not detected	Subtype differentiation and prognosis	Moderate
Yan et al. [44]	Experimental cell-based study	Low (*in vitro*)	Low: rigorous methods	High: no clinical validation	Not applicable	High: *in vitro* only	Not detected	Mechanistic insight into ferroptosis	Low
Sun et al. [39]	Preclinical, bioinformatics, *in vitro*	Low (preclinical)	Low: validated assays	Moderate: cell lines only	Low: consistent across models	High: lacks clinical relevance	Not detected	Chemoresistance link, novel targets	Low
Qian et al. [43]	*In vitro* + *in vivo* experimental study	Low (preclinical)	Low: functional and mechanistic	Moderate: limited animal data	Low: replicated *in vivo*/*in vitro*	Moderate: limited clinical translation	Not detected	Novel ATF3-mediated mechanism	Low to Moderate
Li et al. (2023) [48]	*In vitro*, *in silico*, transcriptomic, IHC	Low (non-RCT)	Low: well-controlled	High: lacks patient outcome data	Not applicable	Moderate: lacks outcome validation	Not detected	STING–IFN–ferroptosis axis discovery	Low to Moderate
Yang et al. [51]	Bioinformatics on public datasets	Low (bioinformatics)	Moderate: public datasets only	High: no clinical endpoints	Not assessed	High: bioinformatic only	Not detected	High diagnostic potential	Low
Li et al. (2022) [50]	Retrospective bioinformatics + IHC	Low (observational)	Moderate: selection and reporting bias	Moderate: small cohort	Not assessed	Moderate: not externally validated	Possible	Prognostic model + immune impact	Low
Bebber et al. [46]	Preclinical human + mouse models	Low (preclinical)	Low: experimental confirmation	Low: patient + PDX validated	Low: mechanistic consistency	Low: highly translational	Not detected	Multiple death pathways integrated	Moderate
Iida et al. [45]	*In vitro* study on cell lines	Low (preclinical)	Low: consistent method	Moderate: small sample	Low	Moderate: cell models only	Not detected	Targets drug-resistant SCLC	Low
Yang et al. [53]	Pharmaco transcriptomic profiling	Low (computational)	Low: large datasets, curated analysis	Low: broad validation	Low	Moderate: computational model	Possible	New drug prediction and survival link	Moderate
Zhou et al. (2023) [54]	Bioinformatics + Retrospective Clinical Study	Low (observational)	Moderate: possible selection bias	Moderate: n = 105	Not assessed (single clinical cohort)	Moderate: not focused on SCLC exclusively	Not detected	Large effect size (AUC > 0.70)	Moderate
Li et al. (2024) [40]	Bioinformatics + IHC validation in LUAD	Low (observational)	Moderate: limited validation	High: lacks functional confirmation	Not applicable	High: LUAD-focused, not generalizable to SCLC	Not detected	Identifies FKBP3 as novel immune /ferroptosis marker	Low
Liu et al. (2025) [56]	Experimental (*in vitro* & *in vivo*)	Low	Low: well-controlled preclinical study	High: small sample (n = 5/group typical)	Not assessed (preclinical)	Moderate: non-human models	Not detected	Mechanistic insight; dual targeting approach with clinical relevance	Low to Moderate
Liu et al. (2024) [41]	Experimental lab study (*in vitro* & clinical tissues)	Low	Moderate: lack of replication across multiple models	Moderate: limited validation in diverse models	Not assessed	Moderate: no direct link to ferroptosis mechanism	Not detected	Suggests novel therapeutic target; not directly linked to ferroptosis	Low
Johnson et al. (2024) [57]	Preclinical *in vitro* & *in vivo*	Low	Low: robust *in vitro*/*in vivo* correlation	Moderate: small xenograft size	Not assessed	Low: translational relevance	Not detected	Potential for repurposing approved drug (Auranofin)	Moderate
Nie et al. (2023) [58]	*In vitro* cell culture study	Low	Moderate: single cell line use	High: limited model variety	Not assessed	Moderate: lacks *in vivo* confirmation	Not detected	Autophagy-ferroptosis link; promising nanodelivery platform	Low
Simbolo et al. (2022) [49]	Transcriptomic /genomic analysis	Low	Moderate: small sample (n = 13)	High: rare subtype focus	Not assessed	Moderate: indirect ferroptosis inference	Possible	Defines molecular subtypes; therapeutic implications	Low
Otsuki et al. (2020) [59]	*In vitro* & *in vivo*	Low	Moderate: not all findings validated *in vivo*	Moderate	Not assessed	Low: relevant to human cancer	Not detected	Explores ferroptosis resistance mechanisms	Moderate
Bi et al. (2019) [42]	Experimental (*in vitro* & *in vivo*)	Low	Moderate: limited sample reporting	Moderate	Not assessed	Low: SCLC included among cancers	Not detected	Links MTDH to ferroptosis; potential target	Moderate

Note: GRADE, Grading of Recommendations, Assessment, Development and Evaluation; ATF3, Activating Transcription Factor 3; RCT, Randomized Controlled Trial; STING, Stimulator of Interferon Genes; IFN, Interferon; IHC, Immunohistochemistry; LUAD, Lung Adenocarcinoma; SCLC, Small Cell Lung Cancer; FKBP3, FK506 Binding Protein 3; MTDH, Metadherin.

## 3 Results

We have subdivided the section Results into five thematic subsections to provide an in-depth, systematically organized synthesis of the molecular mechanisms underlying ferroptosis in SCLC.

### 3.1 Regulatory Pathways and Genetic Determinants of Ferroptosis in SCLC

The molecular landscape of SCLC, defined by nearly universal inactivation of TP53 and RB1 and dominated by one of four key transcription factors (ASCL1, NEUROD1, POU2F3, YAP1), creates a unique context for ferroptosis regulation [1]. The core machinery of ferroptosis, centered on the balance between pro-death lipid peroxidation (driven by ACSL4, iron) and antioxidant defense (mediated by GPX4/GSH, FSP1/CoQ10), is profoundly influenced by these SCLC driver pathways. The subsequent sections will analyze how specific molecular players, including HMOX1, FKBP3, PTPMT1, and MTDH, function as critical nodes within this SCLC-ferroptosis interface. The role of heme oxygenase-1 (HMOX1) in regulating ferroptosis sensitivity in SCLC has been evaluated by Sun and Zhang [39]. Functional analysis demonstrates that HMOX1 is downregulated in drug-resistant SCLC cell lines, while its upregulation enhances chemosensitivity, promotes lipid peroxidation, and increases the expression of ferroptosis-associated proteins (ACSL4, CD71, transferrin, ferritin heavy and light chains) and suppresses GPX4 and xCT, both known ferroptosis inhibitors. HMOX1 knockdown had the opposite effects, decreasing ferroptosis and increasing drug resistance. Mechanistically, the study found that HMOX1 downregulates mic14, a miRNA that acts as a negative regulator of ferroptosis in SCLC cells and mediates chemoresistance by suppressing lipid peroxidation. These findings highlight HMOX1 as a potential therapeutic target to overcome drug resistance and improve treatment outcomes in SCLC. FK506 binding protein 3 (FKBP3) has been identified as a key contributor to lung adenocarcinoma (LUAD) progression through dual mechanisms involving ferroptosis regulation and immune infiltration suppression by Li et al. [40]. By integrating transcriptomic data from public datasets with ferroptosis-related gene sets (GeneCard), the authors identified 184 genes associated with both ferroptosis and LUAD prognosis. Among these, FKBP3 emerged as a top candidate, exhibiting significantly elevated expression in tumor tissues and correlating with reduced overall survival. At the molecular level, FKBP3 was shown to participate in pathways related to DNA replication and repair, cell cycle checkpoints, and epigenetic regulation, interacting with central hub proteins such as HDAC1, HDAC2, MTOR, and PIN1. Gene set enrichment analysis further implicated FKBP3 in TP53-regulated transcription, ubiquitin-mediated proteolysis, and endoplasmic reticulum stress, suggesting its involvement in oxidative stress response and proteome remodelling. Additionally, FKBP3 expression was found to be positively associated with pro-tumorigenic Th2 and T helper cells, while negatively correlated with antitumor immune populations, including CD8^+^ T cells, NK cells, and dendritic cells. The role of Protein Tyrosine Phosphatase Mitochondrial 1 (PTPMT1) in human SCLC cell lines has been investigated, to evaluate PTPMT1’s impact on cell survival and mitochondrial function, by Liu et al. [41]. PTPMT1 knockdown (KO) was induced by lentivirus-mediated short-hairpin RNA (shRNA) transduction and PTPMT1 inhibition (alexidine dihydrochloride). Transcriptome sequencing and untargeted metabolomics were performed to identify dysregulated genes and metabolites. The data showed that PTPMT1 inhibition significantly reduced cell proliferation, colony formation, and migration, while increasing apoptosis. Mitochondrial function was disrupted, as evidenced by decreased JC-1 staining. The transcriptome analysis revealed that pathways related to the respiratory chain and mitochondrial membrane proteins were disrupted. The study linked PTPMT1 inhibition to ferroptosis by testing the ferroptosis inhibitor Fer-1, which did not reverse cell death, suggesting PTPMT1’s role is independent of ferroptosis. Immunohistochemical staining revealed significantly higher PTPMT1 expression in SCLC tissues compared to normal tissues. Survival analysis indicated that PTPMT1 overexpression correlated with poorer prognosis in lung cancer patients. Bi et al. investigated the role of metadherin (MTDH) in modulating ferroptosis sensitivity to various ferroptosis inducers across multiple cancer cell lines, including SCLC [42]. Their data demonstrated that MTDH knockout significantly increased resistance to GPx4 inhibitors, confirming MTDH’s role in promoting ferroptosis susceptibility. Mechanistically, MTDH downregulated GPx4 and SLC3A2 at both mRNA and protein levels, reducing intracellular GSH and increasing lipid peroxidation. Combination therapies (e.g., ML162 + sorafenib) showed enhanced cytotoxicity in MTDH-high cells, suggesting synergistic potential. To confirm *in vitro* experiments, Bi et al. employed an orthotopic breast cancer model using immunodeficient NSG mice implanted with MTDH wild-type (WT) or KO MDA-MB-231 cells. The study aimed to assess whether MTDH-dependent ferroptosis sensitivity could be exploited therapeutically *in vivo*. Mice received intratumoral injections of GPx4 inhibitors (RSL3; ML162) and/or oral sorafenib. Key results revealed that RSL3 + sorafenib combination therapy significantly suppressed tumor growth in MTDH-high xenografts, while MTDH KO tumors were resistant. Immunohistochemistry confirmed reduced GPx4 and SLC3A2 expression in MTDH WT tumors, aligning with *in vitro* data.

### 3.2 Therapeutic Induction of Ferroptosis via Natural Compounds and Pharmacological Agents

Recent evidence has highlighted the potential of natural compounds to modulate ferroptosis in SCLC, a malignancy characterized by poor prognosis and limited therapeutic options. Among these, shikonin, *p*-hydroxy cinnamaldehyde (CMSP), sulforaphane, and total coumarins from *Pileostegia tomentella* (TCPT) have demonstrated significant preclinical efficacy.

Notably, Qian et al. (2023) investigated the anti-cancer effects of shikonin, a naphthoquinone derivative extracted from *Lithospermum erythrorhizon*, demonstrating for the first time its role in inducing ferroptosis in SCLC cells [43]. Mechanistically, shikonin treatment resulted in increased accumulation of ROS and lipid peroxidation, depletion of GSH, and downregulation of GPX4. Central to this mechanism was the upregulation of Activating Transcription Factor 3 (ATF3), which was shown to be essential for ferroptosis induction. Further analysis revealed that shikonin enhanced ATF3 expression via epigenetic modulation—specifically, by inhibiting c-MYC-mediated recruitment of histone deacetylase 1 (HDAC1) to the ATF3 promoter, thereby promoting histone acetylation. *In vivo* validation using a murine xenograft model confirmed the ferroptosis-dependent anti-tumor efficacy of shikonin.

Yan et al. (2025) investigated the antineoplastic mechanism of CMSP in SCLC cell lines, focusing on its ability to induce ferroptosis [44]. Mechanistically, CMSP induces hallmark features of ferroptosis, including elevated ROS, Fe^2+^, and malondialdehyde (MDA) levels, and increased expression of TFR1 and DMT1, along with reduced GSH, SLC7A11, and GPX4 levels. Additionally, CMSP triggers mitochondrial dysfunction, evidenced by decreased mitochondrial volume and membrane potential, increased mitochondrial ROS, and condensed mitochondrial membranes. Importantly, Mitochondrial ROS scavenging (Mito-TEMPO) attenuates CMSP-induced ferroptosis by 50% (*p* = 0.03), implicating mitochondrial dysfunction as a nodal point. CMSP induces HMOX1 overexpression, a known regulator of oxidative stress and ferroptosis. Immunohistochemical analysis revealed lower HMOX1 expression in SCLC tumor tissues compared to adjacent normal tissue, supporting its relevance in disease pathology.

Recent investigations by Iida et al. (2021) have indicated sulforaphane, a dietary isothiocyanate, as a potent ferroptosis inducer in SCLC through modulation of the SLC7A11 axis [45]. In this study, sulforaphane treatment significantly reduced the viability of SCLC cells: Mitochondrial ROS scavenging (Mito-TEMPO) attenuates CMSP-induced ferroptosis by 50% (*p* = 0.03), implicating mitochondrial dysfunction as a nodal point line by downregulating the expression of SLC7A11. The anti-tumor effect of sulforaphane was reversed upon supplementation with ferroptosis inhibitors such as ferrostatin-1, confirming the specificity of the ferroptotic pathway. Furthermore, sulforaphane-induced cell death was more pronounced in non-neuroendocrine (non-NE) SCLC phenotypes, aligning with previous data that suggest heightened ferroptosis susceptibility in non-NE subtypes. These findings propose sulforaphane as a promising ferroptosis-inducing agent and underscore the therapeutic relevance of targeting SLC7A11 in ferroptosis-sensitive SCLC.

At present, no FDA-approved agent is specifically indicated as a ferroptosis inducer for SCLC. Nevertheless, several approved drugs—including sorafenib, sulfasalazine, altretamine, and auranofin—exert ferroptosis-promoting effects via inhibition of system xCT, GPX4, or thioredoxin reductase pathways. These compounds represent viable repurposing candidates, provided their pharmacokinetics and target engagement are validated in SCLC-specific settings

### 3.3 Subtype-Specific Susceptibility and Redox Pathway Interdependence

One of the key discoveries in ferroptosis biology is the differential susceptibility of SCLC molecular subtypes. In a recent study by Bebber et al., the role of phenotypic heterogeneity in SCLC was explored with a specific focus on susceptibility to ferroptosis [46,47]. The authors demonstrated that non-neuroendocrine (non-NE) SCLC subtypes exhibit high sensitivity to ferroptosis, primarily due to subtype-specific remodeling of the lipidome. This vulnerability is linked to elevated expression of ACSL4 and LPCAT3 and increased biosynthesis of ether-linked polyunsaturated phospholipids (PUFAs), which are known to undergo lethal peroxidation during ferroptosis. Genetic and pharmacological experiments confirmed that GPX4 inhibition or cystine deprivation effectively triggered ferroptotic cell death in non-NE cells. Conversely, NE SCLC subtypes are resistant to ferroptosis, despite low glutathione levels, due to selective reliance on the thioredoxin (TRX) antioxidant pathway. NE cells exhibited increased expression of TXNIP and sensitivity to thioredoxin reductase inhibition (e.g., Auranofin). This selective addiction to TRX rendered NE cells vulnerable to redox stress when the pathway was pharmacologically disrupted. Importantly, the study showed that intra-tumoral heterogeneity and plasticity between NE and non-NE states contribute to treatment resistance. Upon targeting a single pathway (either ferroptosis or TRX), the tumor composition shifted toward the resistant phenotype. However, combined inhibition of ferroptosis and the TRX pathway led to synergistic killing of both NE and non-NE cells, overcoming plasticity and intratumoral heterogeneity in xenografts, genetically engineered mouse models, and patient-derived circulating tumor cell models.

The interplay between stimulator of interferon genes (STING) pathway activity and immune responsiveness in SCLC reveals crucial therapeutic vulnerabilities, particularly in the immunologically “cold” STING-low subtypes that predominate in this cancer type [48]. Molecular stratification has identified three STING-related phenotypes, with STING-high tumors characterized by elevated MHC-I expression, immune infiltration, ferroptosis potential, and EMT activity. In contrast, STING-low tumors exhibit pronounced DNA damage response (DDR) activity, high proliferation, and reduced ferroptosis sensitivity, suggesting mechanisms of immune escape and metabolic adaptation. A dual pharmacological approach using ataxia telangiectasia and Rad3-related protein (ATR) and TOP1 inhibition has been shown to restore type I interferon production and induce the expression of MHC-I genes and pro-inflammatory chemokines (CXCL10, CCL5) in selected STING-low SCLC cell models. Furthermore, ATR and TOP1 inhibition stimulate innate immunity and suppresses neuronal differentiation of NE cells. Nevertheless, this immune reactivation appears subtype-dependent, as other STING-low lines failed to respond similarly, emphasizing the heterogeneity of the neuroendocrine landscape. Transcriptomic analyses further support this dichotomy, confirming that STING-low SCLC aligns with proliferation and immune evasion, while STING-high subtypes are associated with ferroptotic vulnerability and enhanced immunogenicity.

The study by Simbolo et al. investigated the molecular and transcriptomic profiles of combined (C)-SCLCs, composed of SCLC admixed with a non-SCLC component, to identify therapeutically relevant subtypes, with a focus on NE vs. non-NE phenotypes and their association with ferroptosis sensitivity [49]. The cohort included three different subtypes based on their non-small-cell component: adenocarcinoma (CoADC), large-cell neuroendocrine carcinoma (CoLCNEC), and squamous cell carcinoma (CoSQC), which were assessed for alterations in 409 genes and transcriptomic profiling of 20,815 genes. Key findings revealed that TP53 and RB1 were the most frequently mutated genes, with additional targetable alterations in KRAS, PIK3CA, and EGFR. Transcriptomic analysis demonstrated that CoLCNEC exhibited a strong NE phenotype (ASCL1/NCAM1-high), while CoSQC displayed a non-NE profile (YAP1-high), and CoADC showed intermediate heterogeneity. Importantly, ferroptosis sensitivity markers (ACSL4, ELOVL5, and SLC7A11) were enriched in non-NE CoSQC and CoADC, whereas NE CoLCNEC was resistant, expressing ferroptosis resistance markers (AIFM2, REST).

### 3.4 Prognostic Relevance of Ferroptosis-Related Gene Signatures

Several prognostic models emphasize the clinical significance of ferroptosis in SCLC.

A recent integrative analysis by Li et al. (2022) developed and validated a prognostic risk-scoring model based on ferroptosis-related genes (FRGs) in SCLC, highlighting their potential utility in clinical stratification and treatment decision-making [50]. By leveraging transcriptomic data from public databases, the authors identified 10 prognostically relevant FRGs using univariate Cox regression and LASSO analyses. The constructed risk model demonstrated robust predictive power for overall survival, effectively classifying patients into high- and low-risk groups with distinct clinical outcomes. Furthermore, functional enrichment analyses revealed that high-risk profiles were associated with pathways linked to cell cycle regulation and DNA replication, while low-risk groups showed enrichment in ferroptosis and immune-related processes. The study also identified several key hub genes, including ALOX5, GPX4, and HMOX1, that may serve as potential therapeutic targets. Importantly, immune infiltration analysis demonstrated significant differences in the tumor microenvironment (TME) between risk groups, suggesting an interplay between ferroptosis susceptibility and immune landscape in SCLC. This work offers a new prognostic framework and supports the integration of ferroptosis-related markers in future precision oncology approaches for SCLC.

Yang et al. (2023) delineated the role of FRGs in SCLC, combining transcriptomic data from multiple public databases to construct a prognostic model based on FRGs expression [51]. The authors identified a panel of FRGs with significant prognostic implications. Unsupervised clustering revealed three molecular subtypes of SCLC; each associated with distinct immune cell infiltration patterns and survival outcomes. A four-gene signature was developed and validated for its prognostic value, showing that high-risk scores correlated with reduced overall survival and increased expression of immunosuppressive markers. Functional enrichment analyses indicated that these genes are involved in oxidative stress response, iron metabolism, and redox homeostasis—hallmarks of ferroptotic processes. Furthermore, the high-risk group displayed features of immune evasion, suggesting potential therapeutic implications for combining ferroptosis induction with immune checkpoint inhibition in SCLC.

Wang et al. (2025) developed a new molecular classification of SCLC based on FRGs expression and TME characteristics [52]. Through transcriptomic and genomic analysis from public datasets, the authors identified three distinct FRG-based subtypes (S1, S2, S3), each associated with unique biological behaviors and clinical implications. Most of the FRGs were predominantly expressed in the S3 subtype, which had the best prognosis. The proposed classification has potential prognostic value and may facilitate precision oncology strategies for SCLC patients.

Recent pharmacotranscriptomic analyses by Yang et al. (2020) have provided critical insights into the gene–drug networks underlying ferroptosis regulation in cancer [53]. In this study, the authors employed large-scale transcriptomic and pharmacological data from multiple cancer types to identify novel compounds and genetic signatures that modulate ferroptosis. Using datasets such as the Cancer Therapeutics Response Portal (CTRP) and the Genomics of Drug Sensitivity in Cancer (GDSC), the study established a ferroptosis score (FerroScore) to quantify ferroptosis sensitivity across cancer cell lines. SCLC showed enrichment of pro-ferroptosis gene signature, suggesting a unique vulnerability of SCLC to ferroptosis inducers. Compounds such as sulfasalazine and RSL3, known inducers of ferroptosis, were validated, and additional candidate drugs with ferroptosis-inducing potential were identified. Moreover, integrative network analyses highlighted several genes, including SLC7A11, GPX4, and ALOX5, as key regulators of ferroptotic cell death. The authors also found that tumors with low FerroScore exhibited resistance to ferroptosis-inducing agents and were characterized by distinct transcriptomic features involving antioxidant defence and iron metabolism.

The study by Zhou et al. explores the role of ferroptosis in the metastatic spread of lung cancer to bone—a common and severe complication in advanced disease [54]. Through a two-phase design combining bioinformatic screening and clinical validation, the authors identified 15 ferroptosis-related genes that were differentially expressed in lung cancer bone metastasis vs. normal lung tissue. These genes were implicated in processes such as oxidative stress response, hypoxia adaptation, iron metabolism, mitochondrial function, and IL-17 signaling. Using public datasets and miRNA interaction analyses, they linked these genes to pathways relevant to tumor dissemination and ferroptotic cell death. Clinical data from 105 lung cancer patients, some with metastasis, revealed that elevated serum ALP and neuron-specific enolase levels were significantly associated with bone metastasis, showing predictive value. While not exclusive to SCLC, the findings are highly relevant to its metastatic biology. Overall, the strength of evidence supporting the prognostic value of ferroptosis-related gene signatures remains moderate to low due to methodological limitations and a lack of clinical validation in most studies. The detailed GRADE assessment is presented in Table 1.

Collectively, these prognostic studies reinforce the subtype-specific nature of ferroptosis in SCLC. A consistent theme emerging from the data is the enrichment of pro-ferroptotic genes like *ACSL4* in non-neuroendocrine subtypes (SCLC-P, SCLC-Y), which correlates with their inherent vulnerability. Conversely, neuroendocrine subtypes (SCLC-A, SCLC-N) frequently exhibit higher expression of antioxidant and anti-ferroptotic genes, such as components of the thioredoxin pathway, underpinning their resistance [46,55]. This dichotomy suggests that optimal therapeutic strategies should be subtype-informed: non-NE tumors may respond robustly to direct GPX4 or SLC7A11 inhibitors, while NE tumors may require priming with TRX pathway inhibitors to sensitize them to ferroptosis inducers, thereby overcoming intrinsic resistance mechanisms.

Table A1 shows the expression of ferroptosis-related genes across the major SCLC molecular subtypes. For each subtype (ASCL1, NEUROD1, POU2F3, YAP1), the table lists predominant pro-ferroptosis genes (e.g., ACSL4, ALOX15, TFRC) and anti-ferroptosis genes (e.g., SLC7A11, GPX4, FSP1), the predicted ferroptosis sensitivity, and supporting references. This addition visually reinforces the textual synthesis in Section 3.4, providing an at-a-glance overview of subtype-specific ferroptotic vulnerabilities and resistance mechanisms, and directly fulfills comparative analysis.

### 3.5 Ferroptosis-Based Combination Therapies and Clinical Translation

Liu et al. investigated the role of PFKFB4 in SCLC and evaluated a biomimetic co-delivery system combining paclitaxel (PTX) and PFKFB4-targeting siRNA (siPFKFB4) in human SCLC cell lines [56]. PFKFB4 knockdown enhanced PTX sensitivity and induced ferroptosis, marked by mitochondrial shrinkage and increased lipid peroxidation. These findings highlight PFKFB4’s role in SCLC chemoresistance and suggest that ferroptosis induction can overcome it. Transcriptomic analysis revealed JAK-STAT pathway activation, linking ferroptosis to immune stimulation. A mouse SCLC xenograft model was established. Treatments included PTX, siPFKFB4, the co-delivery system, and combination with anti-PD-L1. The codelivery system significantly suppressed tumor growth and increased CD8^+^ T cell infiltration. Combination with anti-PD-L1 further enhanced efficacy, suggesting synergy between ferroptosis induction and immunotherapy. Higher PFKFB4 expression levels have been shown in tumors of SCLC patients, correlating with poor prognosis. Johnson et al. investigated the potential of auranofin (AF), an FDA-approved thioredoxin reductase (TRXR) inhibitor, to sensitize SCLC and lung neuroendocrine tumor cells to sorafenib, a multi-kinase inhibitor known to deplete GSH [57]. AF treatment significantly reduced TRXR activity across all cell lines utilized, demonstrating effective target engagement. Sorafenib alone induced dose-dependent GSH depletion, consistent with its known inhibition of the cystine/glutamate antiporter. The combination therapy showed synergistic effects; clonogenic survival was markedly reduced compared to single-agent treatments. These findings highlight the therapeutic potential of simultaneously disrupting both the thioredoxin and glutathione antioxidant systems to induce lethal oxidative stress in SCLC/NET cells. A SCLC xenograft model was developed to determine a safe and efficacious dose of AF that significantly inhibited TRXR in tumors without affecting glutathione peroxidase 1. The median overall survival of the mice with SCLC xenografts was significantly prolonged by treatment with AF. These findings support the hypothesis that AF effectively inhibits TRXR both *in vitro* and *in vivo* in SCLC, sensitizes NETs and SCLC to sorafenib, and could be repurposed as an adjuvant therapy with targeted agents that induce disruptions in thiol metabolism. The clinical relevance is further constrained by using cell line models rather than patient-derived cells, which may better recapitulate tumor heterogeneity. The study by Nie et al. investigated the synergistic effects of iron oxide nanoparticles loaded with paclitaxel (IONP@PTX) on inducing ferroptosis in the human SCLC cell line [58]. IONP@PTX treatment significantly reduced cell viability compared to IONP or PTX monotherapy. It also increased iron levels, ROS, and lipid peroxidation, while modulating autophagy proteins, confirming synergy. The study linked ferroptosis to autophagy activation through the Beclin 1-HDAC6 pathway. Otsuki et al. analysed the potential of the vasodilator oxyfedrine (OXY) to sensitize cancer cells to xCT-targeted therapy by inhibiting aldehyde metabolism, thereby promoting ferroptosis [59]. Multiple human cancer cell lines, including SCLC cell lines, were treated with OXY, the xCT inhibitor sulfasalazine (SSZ) and buthionine sulfoximine (BSO) or combinations of them. Results showed that OXY alone had minimal effect, but combined with SSZ or BSO, it significantly reduced cell viability and increased 4-HNE and ROS levels, linking these effects to ferroptosis via GSH depletion and ALDH inhibition. The study evaluated the efficacy of OXY and SSZ combination therapy *in vivo* using a mouse xenograft model. Mice were treated with saline (control), SSZ, OXY, or both drugs for 16 days. The combination therapy significantly suppressed tumor growth and increased 4-HNE accumulation compared to monotherapies. These findings demonstrated that OXY enhances SSZ-induced ferroptosis in SSZ-resistant cancer cells both *in vitro* and *in vivo*. Microarray analysis of tumor xenograft tissue showed cyclooxygenase-2 expression as a potential biomarker for the efficacy of such combination therapy. Furthermore, OXY-mediated ALDH inhibition was found to sensitize cancer cells to GSH depletion induced by radiation therapy *in vitro*. These findings establish a rationale for repurposing OXY as a sensitizing drug for cancer treatment with agents that induce GSH depletion. Limitations included the use of a single cell line for xenografts and the lack of long-term toxicity data. These findings, summarized in Table 2, consolidate ferroptosis as a multifaceted and clinically actionable vulnerability in SCLC. Genetic, epigenetic, and metabolic regulators converge to determine ferroptosis sensitivity, while intratumoral heterogeneity and immune landscape modulate its therapeutic relevance. Natural and synthetic compounds capable of inducing ferroptosis represent promising therapeutic candidates, particularly when combined with immune checkpoint blockade or redox pathway inhibitors. Importantly, prognostic models based on ferroptosis-related gene signatures offer novel avenues for patient stratification and personalized therapy. Human trials evaluating ferroptosis agents in SCLC cohorts are now warranted.

**Table 2:** Summary of preclinical and transcriptomic studies investigating ferroptosis mechanisms, biomarkers, and therapeutic in SCLC

Study ID/First Author	Study design	Sample size	Model	Biomarkers analyzed	Methods of detection	Outcomes measured	Main findings
Wang et al. [52]	Transcrip tomic analysis, clustering	181 SCLC tumor samples + 34 independent patient cohort	Human tumor tissue (clinical samples)	Ferroptosis-related genes, ASCL1, NEUROD1, POU2F3, YAP1	RNA sequencing, bioinformatic clustering, immune infiltration analysis	Molecular subtype classification, tumor microenvironment characteristics, prognostic outcomes	Identified 3 ferroptosis-based molecular subtypes with distinct prognoses; S2 subtype linked to poor survival and chemoresistance; potential for personalized ferroptosis-targeting therapies.
Yan et al. [44]	Experimental cell-based study	N/A (cell lines)	*In vitro* (SCLC cell lines H1688, SW1271)	GPX4, SLC7A11, TFR1, DMT1, ROS, GSH, MDA, Fe^2+^, HMOX1	Western blot, flow cytometry, fluorescence microscopy, RNAseq, qPCR, immunohistochemistry, biochemical assays	Cell viability, cell death rate, ROS levels, lipid peroxidation (MDA), Fe^2+^ levels, mitochondrial function, gene expression, protein levels	CMSP induces ferroptosis in SCLC cells by promoting mitochondrial dysfunction, increasing ROS and iron levels, and decreasing antioxidant defences (GPX4, SLC7A11, GSH). Ferroptosis is reversible by inhibitors, confirming its role. HMOX1 upregulation is key in this process.
Sun et al. [39]	Preclinical, bioinformatics, *in vitro*	N/A (cell lines and cohorts GSE40275, GSE60052)	Human tissues; SCLC cell lines (drug-sensitive and drug-resistant)	HMOX1, ACSL4, CD71 (TFRC), Transferrin, Ferritin heavy and light chains, GPX4, xCT, mic14	qPCR, Western blot, CCK-8 assay, flow cytometry, lipid peroxidation assay	Chemosensitivity, drug resistance, ferroptosis markers, lipid peroxidation, mic14 expression	HMOX1 downregulated in SCLC and drug-resistant lines; upregulation increases chemosensitivity and ferroptosis via raising ACSL4, CD71, Ferritin and lowering GPX4, xCT; regulates mic14 which inhibits lipid peroxidation and contributes to chemoresistance; overall HMOX1 reverses chemoresistance by promoting ferroptosis.
Qian et al. [43]	Experimental *in vitro* and *in vivo* study	Cell lines SBC-2; xenograft mouse model	*In vitro* (SCLC cell line SBC-2), *In vivo* (xenograft in mice)	ATF3, GPX4, 4-HNE, ROS, GSH	Western blot, flow cytometry, RNA-seq, shRNA knockdown, histone acetylation assays, immunohistochemistry	Cell proliferation, apoptosis, migration, invasion, colony formation, ROS levels, tumor growth	Shikonin suppresses SCLC growth by inducing ATF3-mediated ferroptosis, increasing ROS and lipid peroxidation, decreasing GPX4 and GSH; ATF3 upregulated by histone acetylation; ferroptosis induction essential for anti-tumor effects.
Li et al. (2023) [48]	*In vitro*, *in silico*, transcriptomic, IHC, cohort classification	59 RNA-seq + 62 microarray SCLC samples; 30 FFPE tissue samples	Human SCLC tumors; cell lines (H446, H146, SHP-77, A549)	STING, CASP1, MHC-I, Type I IFN genes, immune checkpoint markers, NE score, EMT score, ferroptosis score	RNAseq, microarray, IHC, ssGSEA, xCell, quanTIseq, WGS, DESeq2, STAR, RSEM	STING subtype classification; immune infiltration; expression of immune genes; response to ATR and TOP1 inhibitors	STING-high SCLC shows high immunogenicity, EMT, and ferroptosis susceptibility; STING-low is immune-cold with high DDR activity; ATR+TOP1 inhibitors reactivate STING–IFN axis in STING-low tumors.
Yang et al. [51]	Bioinformatics analysis of gene expression datasets	Training: 18 SCLC, 18 normal; Test: 12 SCLC, 10 normal	Human gene expression data from GEO datasets (GSE149507, GSE108055)	Six ferroptosis-related genes (ATG3, MUC1, RRM2, IDH2, PARP1, EZH2)	Gene expression microarrays; statistical analyses (LASSO, SVM-RFE, ROC, logistic regression), GSEA, GSVA, CIBERSORT, DGIdb, ceRNA network	Diagnostic accuracy of marker genes; immune infiltration profiling; pathway enrichment; drug target prediction; ceRNA regulatory network	Six FRGs identified as accurate diagnostic markers with high AUC (up to 1.0 combined); genes linked to cell cycle, immune modulation, JAK-STAT and PPAR pathways; MUC1 and PARP1 implicated in immune microenvironment; 40 drugs predicted targeting five marker genes; constructed ceRNA network suggests RNA regulation complexity; validation needed with larger samples and experimental studies.
Li et al. (2022) [50]	Integrative analysis (bioinformatics + retrospective validation + IHC)	77 SCLC patients (cBioPortal dataset) + 20 patients treated with first-line chemo-immunotherapy (IHC validation)	Prognostic risk-scoring model based on ferroptosis-related genes	5 FRGs: CISD1, TXNIP, HILPDA, SLC7A5, SLC2A8)	- RNAseq (cBioPortal) - Cox regression modeling - Immune infiltration (ImmuCellAI) - IHC for TXNIP & PD-L1	Overall survival (OS) - Immune infiltration - Drug sensitivity (IC50) - Drug sensitivity (CCLE + GDSC) - Clinical response to chemo-immunotherapy (iRECIST)	The 5-gene risk score stratified patients into high-/low-risk groups (AUC up to 0.85 at 3 years). TXNIP was correlated with immune infiltration and predicted better prognosis and response to chemo-immunotherapy, outperforming PD-L1 as a biomarker.
Bebber et al. [46]	Experimental study using patient samples, cell lines, GEMMs, CDXs	67 SCLC patient samples (RNA-seq), multiple human and murine cell lines, mouse models, patient-derived xenografts	Human SCLC samples, murine models, human and murine SCLC cell lines, *in vivo* mouse models, CDX models	CASP8, FADD, TRAIL, RIPK1, RIPK3, MLKL, GPX4, SLC7A11 (xCT), ACSL4, LPCAT3, ASCL1, REST1, YAP1, vimentin, TrxR1/TXNRD1, TXNIP	RNA-seq, qPCR, Western blot, lipidomic, mass spectrometry, flow cytometry, CRISPR/Cas9, cell *via*bility assays, MRI, immunohistochemistry	Expression levels of cell death regulators; sensitivity to ferroptosis inducers; cell death via apoptosis, necroptosis, ferroptosis; lipid peroxidation; tumor growth inhibition; survival correlation	Non-NE SCLC sensitive to ferroptosis via ACSL4 and LPCAT3 lipid remodelling; NE SCLC resistant to ferroptosis but dependent on thioredoxin pathway; combined ferroptosis induction and TRX inhibition kills heterogeneous tumors and prevents relapse; low GPX4 and TXNRD1 expression correlates with better survival.
Iida et al. [45]	*In vitro* cell culture study	Multiple human cell lines: NCI-H69, NCI-H82, NCI-H69AR, 16HBE	Human SCLC cell lines and normal bronchial epithelial cells	SLC7A11 (xCT), intracellular Fe^2+^, lipid ROS, GSH levels	RT-qPCR, Western blot, iron assay, flow cytometry (lipid peroxidation, ROS), LDH assay, MTT assay, fluorescence imaging	Cell growth inhibition, cell death (ferroptosis), iron levels, lipid peroxidation, mRNA and protein expression levels	Sulforaphane inhibits SLC7A11 expression, induces ferroptosis characterized by increased iron, lipid ROS, decreased GSH, and cell death in SCLC cells; less cytotoxicity on normal cells; ferroptosis inhibitors and iron chelator block effect; effective also on drug-resistant cells (H69AR).
Yang et al. [53]	Integrated pharmacotranscriptomic analysis correlating drug sensitivity and transcriptomes across solid cancer cell lines	659 solid cancer cell lines; 481 small-molecule drugs	Human cancer cell lines (solid tumors)	Ferroptosis sensitivity and resistance gene signatures, including SLC7A11, GPX4, FSP1 (AIFM2), ZEB1	Transcriptomics (gene expression), drug sensitivity profiling (AUC from concentration-response curves), pharmacogenomics	Drug sensitivity (AUC), ferroptosis sensitivity/resistance, survival outcomes in cancer patients	Identified novel ferroptosis-inducing drug candidates, including HDAC inhibitors; curated gene signatures predicting ferroptosis sensitivity/resistance; SCLC and IDH-mutant gliomas highly sensitive to ferroptosis inducers; HDAC inhibition enhances Erastin-induced ferroptosis; ferroptosis signatures correlated with clinical survival; EMT correlated with ferroptosis sensitivity.
Liu et al. (2025) [56]	Experimental therapeutic study, *in vitro* and *in vivo*	*In vitro*: 2 SCLC cell lines (NCI-H446, DMS114), murine macrophages; *In vivo*: murine SCLC tumor model (RPM cells), n = 5/group typical	Human SCLC cell lines, murine SCLC model, macrophage and dendritic cell cultures	PFKFB4, ferroptosis markers (GPX4, xCT, ACSL4), immune markers (M1/M2 macrophage markers, DC maturation markers, CD8^+^ T cells, cytokines)	IHC, Western blot, flow cytometry, immunofluorescence, TEM, transcriptomics, ELISA, CCK-8 assay, live-dead staining, colony formation	Tumor volume, tumor necrosis, proliferation, ferroptosis induction, immune cell infiltration and activation, cytokine levels, drug sensitivity	Biomimetic co-delivery nanoparticles (siPFKFB4 + paclitaxel) induce ferroptosis in SCLC, enhance paclitaxel sensitivity, promote immune activation (M1 macrophages, DC maturation, CD8^+^ T cells), activate JAK-STAT pathway, and synergize with anti-PD-L1 immunotherapy to suppress tumor growth with minimal toxicity.
Liu et al. (2024) [41]	Experimental laboratory study	Human tissues (SCLC and adjacent normal); Cell lines (H69, A549)	Human tissue samples; *in vitro* cell culture (SCLC cell lines H69, A549)	PTPMT1 protein and mRNA; Glut1, Glut3; FGF21, LAMP3, GDF15, APLN, MT-ND6	Immunohistochemistry, Western blot, qRT-PCR, lentivirus-mediated shRNA knockdown, CCK-8 proliferation assay, colony formation assay, scratch migration assay, flow cytometry (Annexin V apoptosis, JC-1 mitochondrial function), transcriptome sequencing, untargeted metabolomics	PTPMT1 expression levels, cell proliferation, apoptosis, migration, mitochondrial function, transcriptome and metabolomic changes	PTPMT1 is overexpressed in SCLC tissues; its knockdown or inhibition suppresses cell proliferation and migration, induces apoptosis, disrupts mitochondrial function and glucose metabolism (downregulating Glut1 and Glut3); cell death induced is independent of ferroptosis. Transcriptomic and metabolomic analyses confirm disruption of mitochondrial respiratory pathways. PTPMT1 may be a therapeutic target in SCLC.
Johnson et al. (2024) [57]	Preclinical *in vitro* and *in vivo* study	Cell lines: DMS273, DMS53, H727; Mice xenografts: 28 treated, 30 control	*In vitro* human SCLC and NET cell lines; *In vivo* nude mice xenograft model	Thioredoxin reductase (TrxR) activity; glutathione (GSH) levels; glutathione peroxidase 1 (GPx1) activity	Enzymatic assays for TrxR and GPx1 activity; glutathione assay; clonogenic survival assay; ICP-MS for gold in plasma	TrxR activity inhibition; clonogenic survival; tumor growth; survival time; toxicity (CBC, blood chemistry)	Auranofin (AF) inhibits TrxR activity and reduces clonogenic survival in SCLC and NET cells; sensitizes cells to sorafenib by decreasing GSH; 4 mg/kg AF daily in mice significantly inhibits tumor TrxR activity (~75%), prolongs survival from 19 to 23 days, with no systemic toxicity; AF could be repurposed as adjuvant therapy targeting thiol metabolism in SCLC.
Li et al. (2024) [48]	Retrospective bioinformatics + immunohistochemistry (IHC) validation	TCGA-LUAD dataset: 539 LUAD + 59 normal controls; IHC (HPA) on representative tissues	Prognostic analysis of FKBP3 expression; nomogram including clinical parameters	FKBP3 (focus) + top 10 intersecting ferroptosis /prognosis-related genes (ALG3, H2AX, VDAC1, RUVBL2, CISD2, TFAP2A, LDHA, etc.)	- RNA-seq (TCGA) - GeneCards ferroptosis gene set - Cox regression, KM plotter - GO/KEGG, GSEA - PPI (STRING, Cytoscape) - IHC (Human Protein Atlas) - ssGSEA for immune infiltration	- Overall survival (OS) - Diagnostic accuracy (ROC) - Correlation with clinicopathological features - Immune infiltration patterrns	FKBP3 was significantly overexpressed in LUAD vs. normal tissues. High FKBP3 expression was linked to poor OS (multivariate HR = 1.732, *p* < 0.001), advanced pathological stage, and reduced infiltration of CD8^+^ T, NK, and DC cells. FKBP3 promotes LUAD progression via ferroptosis regulation and immunosuppressive microenvironment.
Nie et al. (2023) [58]	*In vitro*, cell culture study	Non specificato	Human tumor cell lines (SCLC NCI-H446, glioblastoma M059K)	GPX4, Nrf2, Beclin1, LC3-II/I, HDAC6, p62, mTORC1	CCK-8 viability assay, ROS fluorescence (DCFH-DA), lipid peroxidation assay (C11-BODIPY), iron quantification, Western blot	Cell growth inhibition, intracellular iron and ROS accumulation, lipid peroxidation, expression of ferroptosis and autophagy markers	IONP@PTX (iron oxide nanoparticles + paclitaxel) synergistically inhibits tumor cell growth vs. PTX alone; induces ferroptosis via autophagy pathway; increases iron, ROS, lipid peroxides; induces autophagic markers; ferroptosis potentiated by autophagy inducer rapamycin; potential therapy for SCLC.
Simbolo et al. (2022) [49]	Integrative molecular analysis (comparative transcriptomic and genomic study)	13 C-SCLC cases	Human	TP53, RB1, KRAS, PTEN, PIK3CA, EGFR; NE/non-NE markers; ferroptosis markers	Next-generation sequencing (NGS), transcriptomic profiling (20,815 genes)	Genomic alterations, lineage classification (NE vs. non-NE), ferroptosis sensitivity	NE and non-NE transcriptional profiles identify therapeutic vulnerabilities in C-SCLC. CoLCNEC = NE/ferroptosis-resistant; CoSQC = non-NE/ferroptosis-sensitive; CoADC = heterogeneous. Targetable mutations (e.g., KRAS G12C, PIK3CA, EGFR) detected.
Otsuki et al. (2020) [59]	*In vitro* + *in vivo* experimental study	NS (multiple cell lines + xenograft)	Human cancer cell lines + mouse xenografts	xCT, GSH, ALDH, 4-HNE, COX-2	Western blot, cytotoxicity assays, microarray, xenograft analysis	Ferroptosis sensitivity, ALDH activity, GSH levels, ROS accumulation, tumor growth	Oxyfedrine sensitizes cancer cells to sulfasalazine-induced ferroptosis by inhibiting ALDH and promoting 4-HNE accumulation; combination therapy overcomes ferroptosis resistance.
Bi et al. (2019) [42]	Experimental study (*in vitro* and *in vivo*)	Not specified	Cancer cell lines and mouse xenografts	GPX4, SLC3A2, MTDH, glutathione, cysteine, glutamate	qPCR, Western blotting, metabolomics, xenograft models	Sensitivity to ferroptosis, glutathione depletion, gene/protein expression	MTDH enhances vulnerability to ferroptosis by suppressing GPX4 and SLC3A2, reducing cysteine and glutathione levels, and increasing sensitivity to ferroptosis inducers.
Zhou et al. (2023) [54]	Bioinformatics analysis + retrospective clinical analysis	105 lung cancer patients (clinical); multiple datasets (bioinformatics)	Human clinical samples + *In silico* (GEO data)	15 ferroptosis-related genes; ALP; NSE	Differential expression analysis from GEO database, miRNA prediction (miRWalk 2.0), miRNA enrichment (miEAA), logistic regression, ROC analysis	Differential gene expression; association between biomarkers (ALP, NSE) and bone stasis risk	Identified 15 ferroptosis-related DEGs in lung cancer with bone metastasis; ALP and NSE are potential serum indicatometars for bone metastasis risk (AUC > 0.70).

Note: ACSL4, Acyl-CoA Synthetase Long Chain Family Member 4; ALDH, Aldehyde Dehydrogenase; ALP, Alkaline Phosphatase; ASCL1, Achaete-scute homolog; ATF3, Activating Transcription Factor 3; Beclin-1, Autophagy-related Protein Beclin-1; CASP1, Caspase 1; CASP8, Caspase 8; CD71/TFRC, Transferrin Receptor; CDX, Circulating Tumor Cell-Derived Xenograft; CISD1, CDGSH Iron Sulfur Domain 1; CMSP, 2-cyano-3-[5-(4-methoxyphenyl)-2-furyl]-N-(5-quinolinyl)-2-propenamide; COX-2, Cyclooxygenase-2; DC, Dendritic Cell; DDR, DNA Damage Response; DMT1, Divalent Metal Transporter 1; EMT, Epithelial–Mesenchymal Transition; EZH2, Enhancer of Zeste Homolog 2; Fe^2+^, Ferrous Iron; FGF21, Fibroblast Growth Factor 21; FSP1, Ferroptosis Suppressor Protein 1; FRG(s), Ferroptosis-Related Gene(s); GDF15, Growth Differentiation Factor 15; GEMM, Genetically Engineered Mouse Model; Glut1/3, Glucose Transporter 1/3; GPX1/GPX4, Glutathione Peroxidase 1/4; GSH, Glutathione; HDAC6, Histone Deacetylase 6; HILPDA, Hypoxia Inducible Lipid Droplet Associated; HMOX1, Heme Oxygenase 1; IHC, Immunohistochemistry; IFN, Interferon; IONP@PTX, Iron Oxide Nanoparticle + Paclitaxel; LC3-II, Microtubule-Associated Protein Light Chain 3-II; LPCAT3, Lysophosphatidylcholine Acyltransferase 3; LUAD, Lung Adenocarcinoma; MDA, Malondialdehyde; micR-14/mic14, microRNA-14; MUC1, Mucin 1; MTDH, Metadherin; MT-ND6, Mitochondrially Encoded NADH Dehydrogenase 6; NE, Neuroendocrine; Nrf2, Nuclear Factor Erythroid 2–Related Factor 2; NSE, Neuron-Specific Enolase; OS, Overall Survival; PARP1, Poly(ADP-ribose) Polymerase 1; PFKFB4, 6-Phosphofructo-2-Kinase/Fructose-2,6-Bisphosphatase 4; PTPMT1, Protein Tyrosine Phosphatase Mitochondrial 1; RIPK1/RIPK3, Receptor-Interacting Protein Kinase 1/3; RRM2, Ribonucleotide Reductase Regulatory Subunit M2; ROS, Reactive Oxygen Species; SCLC, Small Cell Lung Cancer; SLC2A8, Solute Carrier Family 2 Member 8; SLC3A2, Solute Carrier Family 3 Member 2; SLC7A5, Solute Carrier Family 7 Member 5; SLC7A11 (xCT), Solute Carrier Family 7 Member 11 (Cystine/Glutamate Antiporter); SSZ, Sulfasalazine; STING, Stimulator of Interferon Genes; TP53, Tumor Protein p53; TrxR/TXNRD1, Thioredoxin Reductase 1; TXNIP, Thioredoxin-Interacting Protein.

## 4 Discussion

### 4.1 Ferroptosis as a Novel Therapeutic Vulnerability in SCLC

SCLC continues to represent a highly lethal tumor entity, marked by early dissemination, high recurrence, and limited durable response to current therapies [60,61]. Despite modest gains achieved through chemo-immunotherapy regimens, long-term outcomes remain unsatisfactory. In this context, ferroptosis—a regulated cell death process driven by iron-dependent lipid peroxidation—has emerged as a potential Achilles’ heel in SCLC biology [62,63]. Its unique mechanism, distinct from apoptosis or necroptosis, is especially relevant in tumors with high oxidative stress and deregulated iron metabolism [64], features commonly seen in SCLC. Our analysis highlights how ferroptosis susceptibility is tightly linked to molecular subtype-specific features in SCLC. However, it is important to note that the current understanding is skewed towards the neuroendocrine subtypes (SCLC-A, SCLC-N), with fewer studies providing deep mechanistic insights into the POU2F3- and YAP1-driven non-neuroendocrine variants [65]. This imbalance underscores the need for dedicated research on these rarer but therapeutically distinct subgroups to fully exploit ferroptosis across the SCLC spectrum. Non-NE tumors, for example, show enriched polyunsaturated fatty acid content and lipid peroxidation-prone profiles, rendering them more sensitive to ferroptosis. In contrast, NE subtypes depend on redox buffering systems, such as the thioredoxin axis and GPX4 activity, to evade this death mechanism. These findings suggest that ferroptosis may be exploited both therapeutically and diagnostically, providing a molecular rationale for stratified treatment [66].

### 4.2 Ferroptosis and Chemoresistance in SCLC

One of the most pressing clinical issues in SCLC is the development of resistance to platinum-based chemotherapy and emerging targeted agents [67,68]. Recent studies indicate that alterations in ferroptosis-related pathways are not only consequences but also drivers of this resistance [69]. Chemoresistant SCLC cells frequently display upregulation of antioxidant systems including GPX4, SLC7A11, and NRF2, alongside downregulation of pro-ferroptotic regulators such as HMOX1 and ACSL4 [70]. These changes are often reinforced by epigenetic silencing via HDAC1 or microRNA-mediated repression. Importantly, pharmacological induction of ferroptosis has been shown to re-sensitize chemoresistant cells. Agents targeting GPX4 or inhibiting cystine import via xCT restore vulnerability in refractory SCLC models [71]. This positions ferroptosis not merely as an alternative form of cell death but as a strategic modality to overcome therapeutic resistance. Combined regimens that engage ferroptotic machinery may enhance the depth and durability of response in relapsed patients [72].

### 4.3 Immunogenic Effects and Ferroptosis–TIME Interactions

Ferroptosis is both a cytotoxic and immunogenic form of cell death. It triggers the release of damage-associated molecular patterns (DAMPs), lipid mediators (e.g., oxidized phospholipids), and cytokines that can profoundly remodel the TIME. Mechanistically, the release of intracellular content during ferroptosis can activate antigen-presenting cells and promote cross-priming of CD8^+^ T cells. Furthermore, interferon-gamma (IFNγ) from activated CD8^+^ T cells can, in turn, sensitize tumor cells to ferroptosis by downregulating SLC7A11, creating a potent feed-forward loop of anti-tumor immunity [52]. The STING pathway emerges as a key integrator; ferroptotic damage may release mitochondrial DNA, activating the cGAS-STING axis and subsequent type I interferon signaling. These interactions appear subtype-specific; STING-high, non-NE subtypes may be primed for such immunogenic cell death, while STING-low, NE subtypes might require combination therapies to overcome their inherent immune evasion.

Recent studies confirm that ferroptotic lipid peroxidation products activate the cGAS–STING–IFN axis, thereby enhancing CD8^+^ T cell-mediated antitumor immunity in lung adenocarcinoma and SCLC. This new evidence further supports the integration of the STING pathway in our revised discussion as a key immunogenic mechanism linking ferroptosis to anti-tumor immune activation [73]. In SCLC—a tumor with notoriously low immune infiltration—induction of ferroptosis has been associated with increased CD8^+^ T cell recruitment, enhanced antigen presentation, and upregulation of interferon signalling pathways [74]. Notably, combination strategies targeting ferroptosis and immune checkpoints such as PD-1/PD-L1 show synergistic effects in preclinical SCLC models, particularly when coupled with metabolic inhibitors like PFKFB4 antagonists. These results highlight the dual therapeutic potential of ferroptosis, capable of inducing direct tumor cell death while simultaneously potentiating anti-tumor immune responses. This offers an opportunity to convert immune “cold” SCLC tumors into more responsive “hot” ones [75].

### 4.4 Challenges in Clinical Translation

Despite compelling preclinical data, several barriers to clinical implementation of ferroptosis-targeting therapies remain. The first is the lack of validated predictive biomarkers, as current patient selection strategies do not incorporate ferroptosis-related gene expression or lipidomic profiles. Practical clinical integration of ferroptosis biomarkers may proceed through four incremental steps: (1) retrospective assessment of archival tumor biopsies using ACSL4 and GPX4 immunohistochemistry to define positivity thresholds; (2) prospective baseline IHC testing of a minimal FRG panel in trial enrolment biopsies; (3) longitudinal monitoring of plasma lipid peroxidation markers (e.g., malondialdehyde, 4-hydroxynonenal adducts) alongside circulating tumour DNA analyses; and (4) *ex vivo* functional validation using patient-derived organoids (PDOs) when sufficient material is available. Such a tiered strategy is feasible within existing clinical workflows and could expedite biomarker qualification for ferroptosis-directed interventions [52] Additionally, these translational steps are consistent with current FDA and EMA biomarker qualification frameworks [76], which emphasizes co-development of validated biomarkers alongside novel therapeutic agents. This alignment reinforces the clinical feasibility and regulatory relevance of implementing ferroptosis-based companion diagnostics within precision oncology workflows.

Second, the therapeutic induction of ferroptosis carries an inherent risk of on-target, off-tumor toxicity in organs with high iron turnover and metabolic activity, such as the liver, kidneys, and brain. Preclinical models have associated uncontrolled ferroptosis with hepatorenal toxicity and neurodegenerative processes [29]. Therefore, first-in-human trials must incorporate careful dose escalation and dedicated organ-specific monitoring protocols. To maximize the therapeutic index and enable a precision medicine approach, the concurrent development and validation of reliable biomarkers is essential. These could include plasma markers of lipid peroxidation (e.g., isoprostanes), circulating iron parameters, or functional imaging to detect ferroptosis *in vivo*. Tissue-based biomarkers, such as IHC for ACSL4 or GPX4, could stratify patients most likely to respond, thereby minimizing exposure and risk in resistant populations. Several early-phase clinical trials (NCT05649270, NCT06082140) are currently evaluating ferroptosis-inducing agents with systemic toxicity monitoring, which will help define hepatic and neurological safety thresholds and inform translational applications.

**Figure 2:**
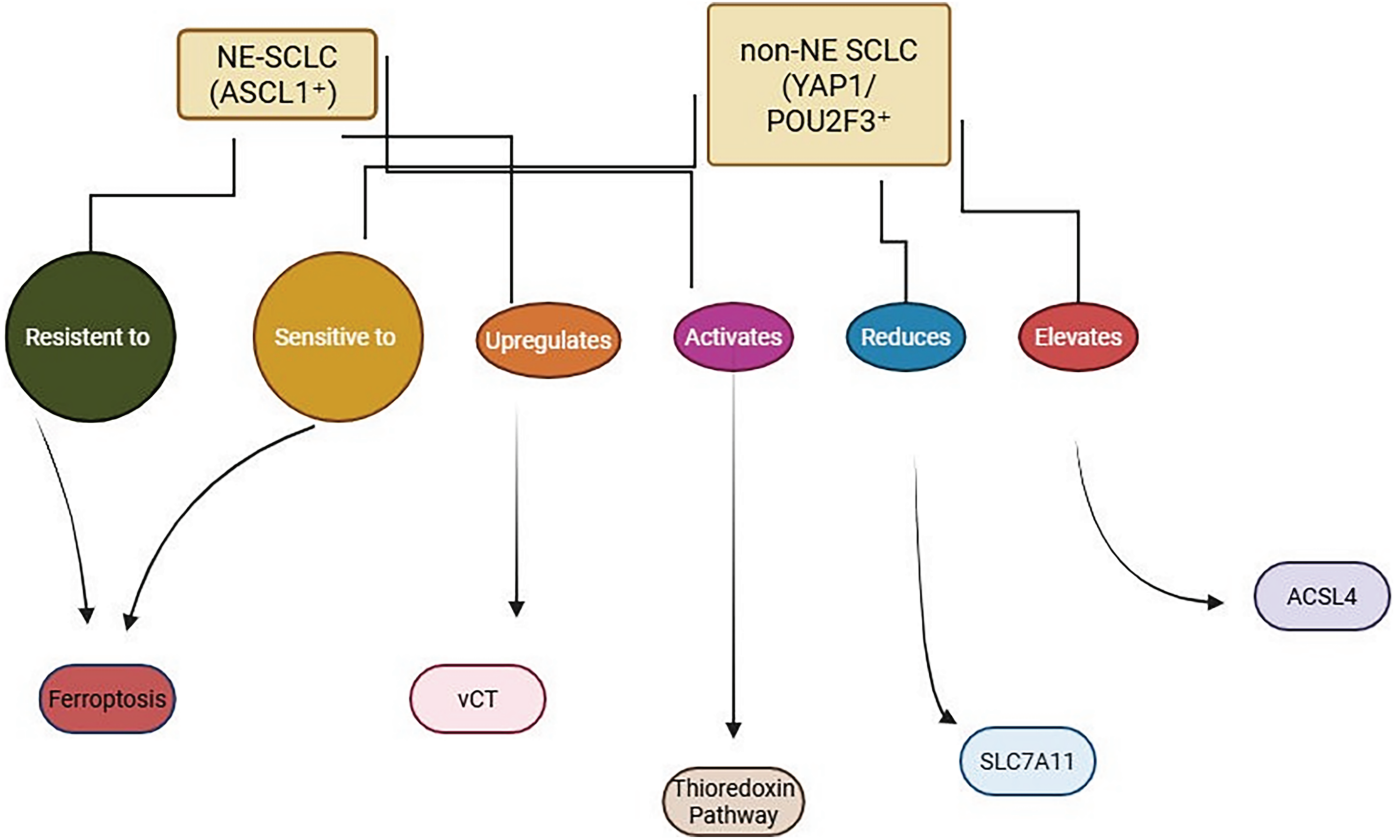
Ferroptosis sensitivity in SCLC subtypes. NE-SCLC (ASCL1^+^) resists ferroptosis via xCT and thioredoxin pathway activation, while non-NE SCLC (YAP1/POU2F3^+^) shows increased ferroptosis sensitivity due to reduced SLC7A11 and elevated ACSL4 expression. BioRender, Toronto, Ontario, Canada (www.biorender.com), July 2025. Note: SCLC, small cell lung cancer; NE-SCLC, neuroendocrine-small cell lung cancer; Non-NE-SCLC, non-neuroendocrine-small cell lung cancer; ASCL1, achaete-scute homolog 1; YAP1, Yes1 associated transcriptional regulator; POU2F3, POU class 2 homeobox 3; xCT, solute carrier family 7 member 11; SCLA11, solute carrier family 7 member 11, ACSL4, acyl-CoA synthetase long chain family member 4

Potential systemic toxicities must be addressed, since ferroptosis affects iron-rich and metabolically active tissues such as the liver and brain, necessitating careful dose escalation and organ-specific monitoring [77]. As organs with high iron turnover or lipid peroxidation susceptibility (particularly the liver and brain) are at risk, systemic ferroptosis induction may result in off-target cytotoxicity and neurotoxicity. Moreover, effector immune cells such as activated CD8^+^ T lymphocytes can themselves undergo ferroptosis, potentially compromising anti-tumour immunity. Excessive lipid peroxidation might also exacerbate inflammatory tissue injury. Therefore, preclinical and clinical development should incorporate comprehensive safety evaluation, including organ histopathology, serum biochemistry, and immune profiling to balance efficacy and tolerability [78–80]. Furthermore, a critical and largely unaddressed question in the current literature is the pharmacokinetic profile of ferroptosis inducers, specifically their ability to penetrate the blood-brain barrier. Given the high frequency of central nervous system metastases in SCLC, the efficacy of these agents against brain lesions is a paramount concern for clinical development [81,82]. Future compounds should be engineered or selected for favorable brain penetration properties [77]. Moreover, adaptive resistance mechanisms may arise as SCLC cells develop escape routes through metabolic rewiring, iron sequestration, or feedback activation of antioxidant networks [64]. To address these challenges, future efforts should prioritize the development of ferroptosis gene signatures for companion diagnostics, integration of multi-omics and single-cell analyses to monitor dynamic changes in ferroptotic susceptibility, and the design of first-in-human trials with ferroptosis inducers, particularly in relapsed or non-NE phenotypes (Fig. 2) [83].

### 4.5 Towards Clinical Translation: Pharmacological Strategies and Research Priorities

The compelling preclinical evidence for ferroptosis induction in SCLC, as systematically reviewed herein, necessitates a focused discussion on translating these mechanistic insights into tangible therapeutic strategies. To address this and provide a clear roadmap, we have synthesized the key molecular vulnerabilities into a framework of actionable pharmacological interventions, as summarized in Table 3 [84].

**Table 3:** Evidence-based ferroptosis-targeting strategies in SCLC

Molecular determinant	Pharmacological strategy	Drugs/Molecules	Reference
SLC7A11 (System Xc^−^)	Direct inhibition	Sulforaphane	[45]
GPX4	Covalent inhibition	RSL3; ML162; Shikonin	[43,46]
FSP1	Enzymatic inhibition	iFSP1	[46]
Thioredoxin pathway	Thioredoxin reductase inhibition	Auranofin	[46,57]
ACSL4	Activation/Sensitization	Mechanistically linked: PPARγ agonists	[46]
Epigenetic modulators	Transcriptional repression of SLC7A11	HDAC Inhibitors; ATR/TOP1 inhibitors	[43,48]
HMOX1	Activation/Induction	Mechanistically linked: HMOX1 inducers	[39]

Note: ACSL4, Acyl-CoA Synthetase Long-Chain Family Member 4; ATR, Ataxia Telangiectasia and Rad3-Related Protein; FSP1, Ferroptosis Suppressor Protein 1; GPX4, Glutathione Peroxidase 4; HDAC, Histone Deacetylase; HMOX1, Heme Oxygenase 1; PPARγ, Peroxisome Proliferator-Activated Receptor Gamma; SCLC, Small Cell Lung Cancer; SLC7A11, Solute Carrier Family 7 Member 11; TOP1, Topoisomerase 1.

This framework highlights a path forward that prioritizes targeted strategies over non-specific approaches. In this context, it is crucial to temper the preclinical enthusiasm for pleiotropic agents with clinical reality. For instance, auranofin was evaluated in a Phase I trial (NCT01737502) in combination with sirolimus for advanced NSCLC and SCLC. The study concluded that while the combination was tolerable, it demonstrated minimal antineoplastic activity, with no objective responses observed in the SCLC cohort [85]. This result underscores the editor’s concern and highlights that the future of targeting the thioredoxin pathway in SCLC may not lie with auranofin itself, but rather with more potent and selective TRX pathway inhibitors or its rational use in biomarker-selected populations (e.g., NE-subtypes with demonstrated TRX addiction) as part of combination regimens [86].

Therefore, the most promising translational path involves a dual approach: the rational repurposing of approved drugs with a clear mechanistic link (e.g., sulforaphane, PPARγ agonists) and the development of next-generation, targeted agents (e.g., brain-penetrant GPX4 inhibitors, FSP1 inhibitors) designed from the outset to overcome the pharmacokinetic and efficacy limitations of first-generation compounds [87]. Beyond the core regulators like GPX4 and FSP1, other pathways offer additional therapeutic leverage. For instance, inhibition of dihydroorotate dehydrogenase (DHODH), a mitochondrial enzyme involved in pyrimidine synthesis, has recently been shown to synergize with GPX4 inhibition to induce ferroptosis, particularly in cells resistant to single-agent therapy [88]. This represents a promising, yet unexplored, combinatorial avenue for SCLC.

To translate ferroptosis from a compelling concept to a clinical reality, specific implementation paths are required. A cornerstone strategy to overcome resistance driven by tumor heterogeneity and phenotypic plasticity is the use of rational combination therapies that simultaneously target multiple, non-redundant redox pathways (e.g., GPX4 inhibition with TRX pathway blockade, as demonstrated by Bebber et al.) [46]. Furthermore, clinical development must be biomarker-driven from the outset, enrolling patients based on molecular subtype (e.g., non-NE with high ACSL4) or validated ferroptosis-related gene signatures. The parallel development of these biomarkers as companion diagnostics is a critical and non-negotiable component of this translational effort. In conclusion, ferroptosis is more than a novel cell death modality—it is a multifaceted platform for therapeutic innovation in SCLC. Its potential to reverse resistance, augment immunotherapy, and redefine molecular stratification could fundamentally improve outcomes in this historically intractable disease.

### 4.6 Leveraging Public Databases for Future Drug Discovery

Beyond repurposing existing drugs, the future of targeting ferroptosis in SCLC lies in the rational design of novel, highly specific agents. The molecular determinants reviewed here provide a rich pipeline of druggable targets (e.g., GPX4, FSP1, ACSL4) [89]. As correctly highlighted, a critical next step is to leverage the biological and biochemical data from public chemical and pharmacological databases to identify lead compounds and optimize chemical probes. Resources such as ChEMBL for bioactivity data, DrugBank for drug-target interactions, and the NCI ALMANAC for drug combination synergy screens can be systematically mined. For instance, researchers can identify existing compounds with predicted activity against ferroptosis targets using structure-based virtual screening; analyze the NCI ALMANAC database to discover synergistic drug pairs that could be harnessed for combination therapies (e.g., an FDA-approved drug that synergizes with a ferroptosis inducer); exploit chemical scaffolds from these databases as starting points for medicinal chemistry optimization to develop more potent and selective GPX4 degraders, FSP1 inhibitors, or ACSL4 activators with improved pharmacokinetic properties [90].

Integrating these computational and database-driven approaches with robust preclinical validation in SCLC-specific models will be essential to translate the biology of ferroptosis into a new generation of clinical candidates [91].

### 4.7 Towards Clinical Translation: Concrete Pharmacological Strategies

Although the systematic review focused on mechanistic studies, we provide here a narrative pharmacological overview of emerging ferroptosis-targeting strategies, not derived from the included studies but based on coherent biochemical pathways discussed throughout the review. This narrative section aims to bridge mechanistic insights with potential translational interventions, offering context for therapeutic development. Ferroptosis represents a promising vulnerability in SCLC, a malignancy historically resistant to standard therapies and characterized by rapid relapse and poor prognosis [92]. Recent advances in redox biology and cancer metabolism have paved the way for pharmacological strategies aimed at modulating key nodes of the ferroptotic pathway. These include inhibitors of the glutathione peroxidase GPX4, blockers of the cystine/glutamate antiporter system Xc^−^, and agents that suppress compensatory antioxidant defenses such as FSP1 and NRF2. In this section, we outline the principal molecular targets implicated in ferroptosis and review current and emerging pharmacological agents under investigation in preclinical or translational contexts [93–95].

### 4.8 Targeting GPX4: Disabling the Master Lipid Antioxidant

Glutathione peroxidase 4 (GPX4) is a selenoenzyme that reduces lipid hydroperoxides to non-toxic lipid alcohols, thus preventing lethal lipid peroxidation. Pharmacological inhibition of GPX4 directly initiates ferroptotic cell death, particularly in cancer cells with high basal oxidative stress.

Among FDA-approved drugs, altretamine (hexamethylmelamine), originally used for ovarian cancer, has been identified as a direct GPX4 inhibitor, inducing ferroptosis without significantly depleting GSH. This suggests a repurposing potential in SCLC, although clinical application in this setting remains investigational [96].

More selectively, RSL3 binds covalently to the selenocysteine active site of GPX4, irreversibly inhibiting its activity. RSL3 has demonstrated robust ferroptotic induction in SCLC cell lines and preclinical synergy with multi-kinase inhibitors such as sorafenib. Other synthetic inhibitors, including ML162 and ML210, exhibit micromolar potency and serve as chemical prototypes in drug optimization campaigns [97].

Natural compounds have also emerged as indirect GPX4 disruptors. Withaferin A, a withanolide derived from *Withania somnifera*, promotes iron-dependent lipid ROS accumulation by inactivating GPX4 and increasing intracellular labile iron. Similarly, FINO2, a peroxide-based molecule, simultaneously oxidizes ferrous iron and indirectly inactivates GPX4, leading to catastrophic lipid peroxidation. Although these compounds are preclinical, they exemplify the chemical diversity in targeting this axis [98].

#### 4.8.1 System Xc^−^ Inhibitors: Starving the Antioxidant Defense

The cystine/glutamate antiporter, known as system Xc^−^, is composed of SLC7A11 (xCT) and SLC3A2. By importing cystine for GSH synthesis, it sustains intracellular antioxidant capacity. Its inhibition depletes GSH and sensitizes cells to ferroptosis.

The anti-inflammatory drug SSZ inhibits SLC7A11 and has shown selective cytotoxicity in SCLC cell lines, which often express xCT at low levels. Studies reveal that SSZ can resensitize resistant xenografts to chemotherapy by amplifying oxidative stress. This drug, already FDA-approved, is now being explored in combinatorial regimens for ferroptosis induction [99].

Erastin, a canonical ferroptosis inducer, similarly inhibits SLC7A11, triggering cystine deprivation and GSH depletion. While not clinically developed due to stability concerns, its derivatives (e.g., imidazole ketone erastin (IKE) are under investigation. Additionally, sorafenib, a clinically approved multi-kinase inhibitor, exhibits off-target activity against xCT and may potentiate ferroptosis when combined with GPX4 inhibitors [100,101].

A novel enzymatic approach involves PEGylated cyst(e)inase, which degrades extracellular cystine and cysteine, mimicking xCT blockade. Preclinical studies have demonstrated profound GSH depletion and ferroptotic cell death in various tumors, highlighting its potential in SCLC models [102].

#### 4.8.2 Inhibition of FSP1: Disrupting the CoQ10 Salvage Axis

FSP1, formerly AIFM2, acts independently of GPX4 to regenerate reduced Coenzyme Q10 (CoQ10-H_2_), a key lipid antioxidant. Blocking FSP1 removes this parallel protective mechanism, particularly effective when GPX4 is concurrently inhibited.

iFSP1, a first-in-class selective inhibitor, impairs FSP1-mediated CoQ10 regeneration, synergizing with GPX4 blockade to induce ferroptosis. Another compound, IMP-1088, interferes with FSP1 myristoylation and membrane localization, indirectly suppressing its activity. While both agents remain preclinical, their combined targeting with GPX4 inhibitors holds promise in overcoming ferroptosis resistance in SCLC [103,104].

#### 4.8.3 NRF2 Inhibitors: Stripping the Antioxidant Shield

NRF2 is a master transcription factor regulating cellular redox homeostasis. In SCLC, mutations in KEAP1 or persistent oxidative stress can lead to constitutive NRF2 activation, enhancing resistance to ferroptosis by upregulating GPX4, SLC7A11, ferritin, and glutathione-related enzymes.

Brusatol, a natural compound, promotes proteasomal degradation of NRF2, while ML385, a synthetic molecule, blocks NRF2 binding to antioxidant response elements. Both agents sensitize tumor cells to ferroptosis and have shown additive effects with GPX4 or xCT inhibitors in preclinical lung cancer models.

Conversely, natural inhibitors such as ginkgetin and isoorientin suppress NRF2 signaling and enhance lipid ROS accumulation. These molecules, though not yet clinically validated, reflect the feasibility of targeting transcriptional regulators of ferroptosis [105–107].

#### 4.8.4 HO-1 and Iron Modulators: Leveraging Iron Overload

Heme oxygenase-1 (HO-1, encoded by HMOX1) catalyzes the degradation of heme into biliverdin, CO, and ferrous iron (Fe^2+^), thereby modulating iron availability and redox tone. While the antioxidant effects of biliverdin/bilirubin may protect normal cells, iron release via HO-1 can trigger ferroptosis under stress. Hemin (hemin arginate), an FDA-approved drug for porphyria, induces HO-1 and promotes ferroptosis via iron overload in cancer cells. In preclinical models of lung cancer, hemin sensitized tumor cells to radiation while sparing normal tissue—likely through selective bilirubin production and ferritin upregulation. This dual effect supports its investigation as a radiosensitizer or adjunct to ferroptosis inducers in SCLC.

Conversely, ZnPP and SnPP, metalloporphyrin inhibitors of HO-1, reduce iron release and suppress ferroptosis. These findings reinforce the pro-ferroptotic role of HO-1 and suggest that its pharmacological activation, rather than inhibition, may benefit therapeutic strategies [108,109].

#### 4.8.5 Clinical Outlook

Despite the preclinical promise, no ferroptosis-specific drugs have been approved for SCLC to date. Nonetheless, repositioned agents such as sulfasalazine, sorafenib, altretamine, and hemin offer clinically actionable entry points. Trials are ongoing to evaluate ferroptosis induction in solid tumors, including in combination with radiotherapy, chemotherapy, and immune checkpoint inhibitors (e.g., anti-PD-1/PD-L1), where ferroptotic stress may enhance immunogenicity. Given the aggressive nature of SCLC and its rapid development of chemoresistance, pharmacological induction of ferroptosis represents an innovative avenue. Future directions will likely involve rational drug combinations targeting multiple ferroptotic regulators (e.g., GPX4 + xCT + FSP1) alongside agents that modulate iron homeostasis. Such multipronged strategies may be essential to overcome cellular redundancy and achieve durable tumor control [62,110].

### 4.9 Limitations and Future Directions

Ferroptosis, a distinct form of regulated cell death characterized by iron accumulation and lipid peroxidation, is increasingly recognized as a promising therapeutic target in SCLC. This neuroendocrine malignancy, which is highly aggressive and refractory to conventional treatments, exhibits substantial intratumoral heterogeneity and metabolic plasticity—factors that contribute to its dismal prognosis. Recent studies have explored the molecular landscape of ferroptosis in SCLC, shedding light on subtype-specific vulnerabilities, redox imbalances, immuno-metabolic crosstalk, and the prognostic significance of ferroptosis-related genes. A primary limitation of the current evidence base is its heavy reliance on conventional cell lines and xenograft models. While these models have been instrumental in identifying core mechanisms, they often fail to fully recapitulate the complex heterogeneity of human SCLC tumors, including critical stromal interactions, the immune contexture, and the dynamic clonal architecture [111–113]. The lack of systematic validation in more physiologically relevant models, such as PDOs and immunocompetent genetically engineered mouse models (GEMMs), represents a significant translational gap that must be addressed to enhance the clinical predictive value of preclinical findings. Another limit is represented by the overrepresentation of NE subtype: only 4 studies profiled POU2F3/YAP1-dominant tumors, skewing mechanistic insights; pharmacokinetic blind spots: no study evaluated blood-brain barrier penetration of ferroptosis inducers, despite SCLC’s frequent CNS metastasis.

To bridge preclinical promise to Phase I/II trials, we propose: 1) Model Systems: prioritize PDOs and immunocompetent GEMMs to test combinations (e.g., auranofin + anti-PD-L1) in subtype-defined contexts; 2) Biomarker-Driven Trials: validate ACSL4 IHC or lipidomic signatures (e.g., PUFA/PE ratios) as enrichment tools for early-phase studies. 3) Drug Optimization: engineer brain-penetrant GPX4 inhibitors [114,115] to target metastatic niches. Additionally, limitations inherent to the review process itself should be acknowledged. Despite adherence to PRISMA 2020 guidelines and systematic search strategies, potential constraints include language bias (English-only inclusion), limited database scope (primarily PubMed and Scopus), and the exclusion of unpublished data or preprints, which may affect the completeness and contemporaneity of the evidence base.

## 5 Conclusions

In closing, the study of ferroptosis in SCLC exemplifies how deep biological insight can reveal unexpected therapeutic opportunities even in cancers traditionally considered intractable. As this field moves forward, it will be critical to maintain the productive interplay between basic science, translational research, and clinical investigation that has brought us to this point. With continued collaboration and innovation, ferroptosis-based therapies may soon claim their place in the SCLC treatment arsenal, offering new hope where it has long been scarce.

## Supplementary Materials



## Data Availability

Not applicable.

## Supplementary Materials

The supplementary material is available online at https://www.techscience.com/doi/10.32604/or.2025.073045/s1.

## Abbreviations

SCLC: Small cell lung cancer
SCL7A11: Solute carrier family 7 member 11
GPX4: Glutathione peroxidase 4
FSP1: Ferroptosis suppressor protein 1
ACSL4: Acyl-CoA synthetase long chain family member 4
ASCL1: Achaete-scute homolog 1
NEUROD1: Neuronal differentiation 1
POU2F3: POU class 2 homeobox 3
YAP1: Yes1 associated transcriptional regulator
EP: Etoposide-platinum
ES: Extensive stage
LD: Limited disease
TFs: Transcription factors
NE: Neuroendocrine
GSH: Glutathione
ROS: Reactive oxygen species
TIME: Tumor immune microenvironment
NSCLC: Non-small cell lung cancer
WOS: Web of science
GRADE: Grading of recommendations, assessment, development and evaluation
HMOX1: Heme oxygenase-1
FKBP3: FK506 binding protein 3
LUAD: Lung adenocarcinoma
PTPMT1: Protein tyrosine phosphatase mitochondrial 1
KO: Knockdown
ShRNA: Short-hairpin RNA
MTDH: Metadherin
WT: Wild-type
CMSP: *p*-hydroxy cinnamaldehyde
TCPT: *Pileostegia tomentella*
ATF3: Activating transcription factor 3
HDCA1: Histone deacetylase 1
MDA: Malondialdehyde
Non-NE: Non-neuroendocrine
PUFAs: Polyunsaturated phospholipids
TRX: Thioredoxin
DDR: DNA damage response
CoADC: Adenocarcinoma
CoLCNEC: Large-cell neuroendocrine carcinoma
CoSQC: Squamous cell carcinoma
FRGs: Ferroptosis-related genes
TME: Tumor microenvironment
CTRP: Cancer therapeutics response portal
GDSC: Genomics of drug sensitivity in cancer
PTX: Paclitaxel
SiPFKFB4: PFKFB4-targeting siRNA
AF: Auranofin
TRXR: Thioredoxin reductase
OXY: Oxyfedrine
SSZ: Sulfasalazine
BSO: Sulfoximine
DAMPs: Damage-associated molecular patterns
INF-gamma: Interferon-gamma
PDOs: Patient-derived organoids
DHODH: Dihydroorotate dehydrogenase
CoQ10-H2: Coenzyme Q10
Fe^2+^: Ferrous iron
GEMMs: Immunocompetent genetically engineered mouse models

## Appendix A

**Table A1:** Summary of included studies investigating ferroptosis in small cell lung cancer (SCLC)

Author (Year)	Study Design/Model	Key Biomarkers/Targets	Mechanistic Focus	Main Outcome/Finding	Ferroptosis Modulation	Reference
Sun et al. (2024)	Preclinical, *in vitro* /bioinformatics	HMOX1, GPX4, ACSL4, micR-14	Chemoresis tance and redox regulation	HMOX1 upregulation enhances chemosensitivity via ferroptosis activation	↑ Ferroptosis (pro-ferroptotic)	[39]
Li et al. (2022)	Bioinformatic + IHC validation	TXNIP, CISD1, HILPDA	Prognostic ferroptosis gene signature	5-gene model predicts OS and immunotherapy response	Mixed (predictive)	[50]
Yang et al. (2023)	Transcriptomic clustering	ATG3, MUC1, IDH2, PARP1	Prognostic and immune contexture	Ferroptosis-related clusters define immune subtypes	Mixed	[51]
Bebber et al. (2021)	*In vitro*, *in vivo* (PDX, GEMMs)	ACSL4, LPCAT3, GPX4, TXNRD1	Subtype susceptibility (NE vs. non-NE)	Non-NE SCLC is highly ferroptosis-sensitive; NE SCLC depends on thioredoxin axis	↑/↓ (context-dependent)	[46]
Li et al. (2023)	*In silico* + *in vitro* + IHC	STING, CASP1, IFN genes	STING/IFN ferroptosis link	ATR + TOP1 inhibition reactivates ferroptosis-associated immunity	↑ Ferroptosis	[48]
Wang et al. (2025)	Transcriptomic/ clustering	FRG signature	Molecular subtypes by ferroptosis potential	Defines 3 FRG-based SCLC clusters with prognostic relevance	—	[52]
Qian et al. (2023)	*In vitro* + xenograft	ATF3, GPX4, GSH	Shikonin-induced ferroptosis	Histone-acetylation-dependent ATF3 upregulation triggers ferroptosis	↑ Ferroptosis	[43]
Yang et al. (2020)	*In vitro*	SLC7A11, GPX4, TFR1, DMT1	Mitochondrial dysfunction and ROS	CMSP induces ferroptosis via HMOX1-ROS-Fe^2+^ axis	↑ Ferroptosis	[53]
Iida et al. (2021)	*In vitro*	SLC7A11 (xCT)	Dietary compound effects	Sulforaphane suppresses xCT, induces ferroptosis in drug-resistant SCLC	↑ Ferroptosis	[45]
Liu et al. (2025)	*In vitro* + *in vivo*	PFKFB4, GPX4, xCT, ACSL4	Co-delivery nanoplatform (siPFKFB4 + PTX)	Induces ferroptosis, enhances chemo- and immunotherapy	↑ Ferroptosis	[56]
Johnson et al. (2024)	*In vitro* + xenograft	TrxR, GSH, GPX1	Dual redox inhibition (Auranofin + Sorafenib)	Synergistic lethality via GSH/TRX depletion	↑ Ferroptosis	[57]
Nie et al. (2023)	*In vitro*	GPX4, Beclin-1, LC3-II	Nanoparticle-mediated ferroptosis/autophagy	IONP@PTX induces ferroptosis via autophagy	↑ Ferroptosis	[58]
Otsuki et al. (2020)	*In vitro* + xenograft	xCT, ALDH, COX-2	ALDH inhibition + SSZ synergy	Oxyfedrine enhances SSZ-induced ferroptosis	↑ Ferroptosis	[59]
Simbolo et al. (2022)	Comparative transcriptomics (C-SCLC)	TP53, RB1, KRAS, ferroptosis genes	NE vs. non-NE profiles	Non-NE components ferroptosis-sensitive; NE components resistant	Context-dependent	[49]
Zhou et al. (2023)	Bioinformatics + clinical	15 FRGs; ALP; NSE	Bone metastasis signatures	ALP/NSE predict bone spread via ferroptosis-linked pathways	Correlative	[54]
Li et al. (2024)	LUAD dataset + IHC	FKBP3	Ferroptosis–immune cross-talk	FKBP3 overexpression correlates with poor OS and immune suppression	↓ Ferroptosis	[40]
Bi et al. (2019)	*In vitro* + xenograft	MTDH, GPX4, SLC3A2	Ferroptosis sensitization	MTDH promotes ferroptosis by downregulating GPX4	↑ Ferroptosis	[42]
Liu et al. (2024)	*In vitro* + tissue	PTPMT1	Mitochondrial function /apoptosis	PTPMT1 overexpression drives survival independently of ferroptosis	Neutral	[41]
Yang et al. (2020)	Pharmacotran scriptomics	SLC7A11, GPX4, FSP1	Drug–gene interaction mapping	Identifies ferroptosis-inducing drug signatures in SCLC	Predictive	[53]

Note: ACSL4, Acyl-CoA Synthetase Long Chain Family Member 4; ALDH, Aldehyde Dehydrogenase; ALP, Alkaline Phosphatase; ATF3, Activating Transcription Factor 3; ATG3, Autophagy Related 3; Beclin-1, Autophagy-Related Protein Beclin-1; CASP1, Caspase 1; CISD1, CDGSH Iron Sulfur Domain 1; CMSP, 2-cyano-3-[5-(4-methoxyphenyl)-2-furyl]-N-(5-quinolinyl)-2-propenamide; COX-2, Cyclooxygenase-2; DMT1, Divalent Metal Transporter 1; FKBP3, FK506 Binding Protein 3; FRG, Ferroptosis-Related Gene; FRGs, Ferroptosis-Related Genes; FSP1, Ferroptosis Suppressor Protein 1; GEMMs, Genetically Engineered Mouse Models; GPX1, Glutathione Peroxidase 1; GPX4, Glutathione Peroxidase 4; GSH, Glutathione; HILPDA, Hypoxia Inducible Lipid Droplet-Associated; HMOX1, Heme Oxygenase 1; IDH2, Isocitrate Dehydrogenase 2; IFN, Interferon; IHC, Immunohistochemistry; KRAS, Kirsten Rat Sarcoma Viral Oncogene Homolog; LC3-II, Microtubule-Associated Protein 1A/1B-Light Chain 3; LPCAT3, Lysophosphatidylcholine Acyltransferase 3; LUAD, Lung Adenocarcinoma; micR-14, microRNA-14; MTDH, Metadherin; MUC1, Mucin 1; NE, Neuroendocrine; NSE, Neuron-Specific Enolase; OS, Overall Survival; PARP1, Poly (ADP-ribose) Polymerase 1; PDX, Patient-Derived Xenograft; PFKFB4, 6-Phosphofructo-2-Kinase/Fructose-2,6-Biphosphatase 4; PTPMT1, Protein Tyrosine Phosphatase Mitochondrial 1; RB1, RB Transcriptional Corepressor 1; ROS, Reactive Oxygen Species; SCLC, Small Cell Lung Cancer; SLC3A2, Solute Carrier Family 3 Member 2; SLC7A11, Solute Carrier Family 7 Member 11 (xCT); SSZ, Sulfasalazine; STING, Stimulator of Interferon Genes; TFR1, Transferrin Receptor 1; TP53, Tumor Protein p53; TrxR, Thioredoxin Reductase; TXNIP, Thioredoxin Interacting Protein; TXNRD1, Thioredoxin Reductase 1; xCT, Cystine/Glutamate Antiporter (SLC7A11); “↑” Indicates Increased Ferroptosis or Ferroptosis-Sensitizing Effect; “↓” Indicates Suppressed or Decreased Ferroptosis; “—” Indicates no Direct Modulatory Effect or not Assessed.
