# Supplementary material for: Ferroptosis as a Translational Axis in Small Cell Lung Cancer: A Systematic Review of Redox Pathways and Precision Oncology Prospects
Source: Oncol Res. 2026 Mar 23;34(4):5. doi: 10.32604/or.2025.073045 (PMC13040312; doi:10.32604/or.2025.073045)
Supplement: Supplementary file 1 [file OncolRes-34-73045-s001.docx]

| **Section and Topic** | **Item No** | **Checklist item** | **Reported on Page Number/Line Number** | **Reported on Section/Paragraph** |
| --- | --- | --- | --- | --- |
| **TITLE** | | |  |  |
| Title | 1 | Identify the report as a systematic review. | Title page, line1 | Title page, line1 |
| **ABSTRACT** | | |  |  |
| Abstract | 2 | See the PRISMA 2020 for Abstracts checklist. | Structured Abstract page 1 | Structured Abstract page 1 |
| **INTRODUCTION** | | |  |  |
| Rationale | 3 | Describe the rationale for the review in the context of existing knowledge. | Introduction, p. 2,3 | Introduction, Paragraphs 1–2 |
| Objectives | 4 | Provide an explicit statement of the objective(s) or question(s) the review addresses. | End of Introduction page 3 | Introduction, Final Paragraph |
| **METHODS** | | |  |  |
| Eligibility criteria | 5 | Specify the inclusion and exclusion criteria for the review and how studies were grouped for the syntheses. | Methods, Eligibility criteria, page 3,4 | Methods, Section 2.1 Eligibility Criteria |
| Information sources | 6 | Specify all databases, registers, websites, organisations, reference lists and other sources searched or consulted to identify studies. Specify the date when each source was last searched or consulted. | Methods, Eligibility criteria, page 3,4,5 | Methods, Section 2.2 – Search Strategy |
| Search strategy | 7 | Present the full search strategies for all databases, registers and websites, including any filters and limits used. | Methods, Search Strategy page 3,4,5 | Methods, Section 2.2 – Search Strategy |
| Selection process | 8 | Specify the methods used to decide whether a study met the inclusion criteria of the review, including how many reviewers screened each record and each report retrieved, whether they worked independently, and if applicable, details of automation tools used in the process. | Methods, Study selection page 3,4,5 | Methods, Methods, Section 2.3, |
| Data collection process | 9 | Specify the methods used to collect data from reports, including how many reviewers collected data from each report, whether they worked independently, any processes for obtaining or confirming data from study investigators, and if applicable, details of automation tools used in the process. | Methods, Data extraction page 3,4,5 | Methods, Methods, Section 2.4 |
| Data items | 10a | List and define all outcomes for which data were sought. Specify whether all results that were compatible with each outcome domain in each study were sought (e.g. for all measures, time points, analyses), and if not, the methods used to decide which results to collect. | Methods, Outcomes section page 3,4,5 | Methods, |
|  | 10b | List and define all other variables for which data were sought (e.g. participant and intervention characteristics, funding sources). Describe any assumptions made about any missing or unclear information. | Methods, Data extraction page 3,4,5 | Methods, Section 2.4 |
| Study risk of bias assessment | 11 | Specify the methods used to assess risk of bias in the included studies, including details of the tool(s) used, how many reviewers assessed each study and whether they worked independently, and if applicable, details of automation tools used in the process. | Methods, Risk of Bias, page 3,4,5 | Methods, Section 2.4 – Data Extraction and Quality Assessment *(last paragraph)* |
| Effect measures | 12 | Specify for each outcome the effect measure(s) (e.g. risk ratio, mean difference) used in the synthesis or presentation of results. | Methods, Statistical Analysis page 3,4,5 | Methods, Section 2.6 – Statistical Analysis *(first paragraph)* |
| Synthesis methods | 13a | Describe the processes used to decide which studies were eligible for each synthesis (e.g. tabulating the study intervention characteristics and comparing against the planned groups for each synthesis (item #5)). | Methods, Synthesis approach page 3,4,5 | Methods, Section 2.6 – Statistical Analysis *(entire section)* |
|  | 13b | Describe any methods required to prepare the data for presentation or synthesis, such as handling of missing summary statistics, or data conversions. | Methods, Data Preparation page 3,4,5 | Methods, Section 2.6 – Statistical Analysis *(entire section)* |
|  | 13c | Describe any methods used to tabulate or visually display results of individual studies and syntheses. | Methods, Summary tables page 3,4,5 | Methods, Section 2.6 – Statistical Analysis *(entire section)* |
|  | 13d | Describe any methods used to synthesize results and provide a rationale for the choice(s). If meta-analysis was performed, describe the model(s), method(s) to identify the presence and extent of statistical heterogeneity, and software package(s) used. | Not applicable (no meta-analysis) page 3,4,5 | Methods, Section 2.6 – Statistical Analysis *(entire section)* |
|  | 13e | Describe any methods used to explore possible causes of heterogeneity among study results (e.g. subgroup analysis, meta-regression). | Not applicable | Not applicable |
|  | 13f | Describe any sensitivity analyses conducted to assess robustness of the synthesized results. | Not applicable | Not applicable |
| Reporting bias assessment | 14 | Describe any methods used to assess risk of bias due to missing results in a synthesis (arising from reporting biases). | Methods, Bias Assessment page 3,4,5 | Discussion, Section 4.9 – Limitations and Future Directions *(final paragraph)* |
| Certainty assessment | 15 | Describe any methods used to assess certainty (or confidence) in the body of evidence for an outcome. | Not performed | Not performed |
| **RESULTS** | | |  |  |
| Study selection | 16a | Describe the results of the search and selection process, from the number of records identified in the search to the number of studies included in the review, ideally using a flow diagram. | Results, PRISMA Flow Diagram page 6 | Results, Section 3.1 – Study Selection *(including PRISMA Flow Diagram, Figure 1)* |
|  | 16b | Cite studies that might appear to meet the inclusion criteria, but which were excluded, and explain why they were excluded. | Results, page 6-14 | Results, Section 3.1 – Study Selection *(including PRISMA Flow Diagram, Figure 1)* |
| Study characteristics | 17 | Cite each included study and present its characteristics. | Results, Table 1, page 6-14 | Results, Section 3.2 – Study Characteristics *(see also Supplementary Table S1)* |
| Risk of bias in studies | 18 | Present assessments of risk of bias for each included study. | Results, page 6-14 | Results, Section 3.3 – Quality Assessment *(summary of bias domains)* |
| Results of individual studies | 19 | For all outcomes, present, for each study: (a) summary statistics for each group (where appropriate) and (b) an effect estimate and its precision (e.g. confidence/credible interval), ideally using structured tables or plots. | Results, Tables 2–3, , page 6-14 | Results, Section 3.4 – Key Findings from Included Studies *(Tables 1–2)* |
| Results of syntheses | 20a | For each synthesis, briefly summarise the characteristics and risk of bias among contributing studies. | Results, Narrative Summary, , page 6-14 | Results, Section 3.5 – Thematic Synthesis of Ferroptosis Mechanisms |
|  | 20b | Present results of all statistical syntheses conducted. If meta-analysis was done, present for each the summary estimate and its precision (e.g. confidence/credible interval) and measures of statistical heterogeneity. If comparing groups, describe the direction of the effect. | Not applicable | Not applicable |
|  | 20c | Present results of all investigations of possible causes of heterogeneity among study results. | Not applicable | Not applicable |
|  | 20d | Present results of all sensitivity analyses conducted to assess the robustness of the synthesized results. | Not applicable | Not applicable |
| Reporting biases | 21 | Present assessments of risk of bias due to missing results (arising from reporting biases) for each synthesis assessed. | Results, Reporting Bias, page 6-14 | Discussion, Section 4.9 – Limitations and Future Directions *(final paragraph)* |
| Certainty of evidence | 22 | Present assessments of certainty (or confidence) in the body of evidence for each outcome assessed. | Not applicable – No formal GRADE assessment performed in this review | Not applicable – No formal GRADE assessment performed in this review |
| **DISCUSSION** | | |  |  |
| Discussion | 23a | Provide a general interpretation of the results in the context of other evidence. | Discussion, Page 14-24 | Discussion, Paragraphs 1–3 *(sections on therapeutic vulnerability, chemoresistance, and TIME interactions)* |
|  | 23b | Discuss any limitations of the evidence included in the review. | Discussion, Page 14-24 | Discussion, Section 4.9 – Limitations and Future Directions *(first half)* |
|  | 23c | Discuss any limitations of the review processes used. | Discussion, Page 14-24 | Discussion, Section 4.9 – Limitations and Future Directions *(final paragraph)* |
|  | 23d | Discuss implications of the results for practice, policy, and future research. | Discussion, Page 14-24 | Discussion, Paragraphs 3–4 and Section 4.9 – Final paragraph |
| **OTHER INFORMATION** | | |  |  |
| Registration and protocol | 24a | Provide registration information for the review, including register name and registration number, or state that the review was not registered. | Methods, | Methods, Section 2.1 – Eligibility Criteria) |
|  | 24b | Indicate where the review protocol can be accessed, or state that a protocol was not prepared. | Methods, Protocol Section | Methods, Section 2.1 – Eligibility Criteria) |
|  | 24c | Describe and explain any amendments to information provided at registration or in the protocol. | Methods, Protocol Section | Title Page – Acknowledgments section |
| Support | 25 | Describe sources of financial or non-financial support for the review, and the role of the funders or sponsors in the review. | Funding Acknowledgments | Title Page – Acknowledgments section |
| Competing interests | 26 | Declare any competing interests of review authors. | Declaration of Conflicts of Interest | Title Page – Conflict of Interest section |
| Availability of data, code and other materials | 27 | Report which of the following are publicly available and where they can be found: template data collection forms; data extracted from included studies; data used for all analyses; analytic code; any other materials used in the review. | Data Availability Statement | Title Page – Data Availability Statement |

*As the checklist was provided upon initial submission, the page number/line number reported may be changed due to copyediting and may not be referable in the published version. In this case, the section/paragraph may be used as an alternative reference.
